# Supplementary material for: Lack of context modulation in human single neuron responses in the medial temporal lobe
Source: Cell Rep. 2025 Jan 15;44(1):115218. doi: 10.1016/j.celrep.2024.115218 (PMC11781864; doi:10.1016/j.celrep.2024.115218)
Supplement: Document S1. Figures S1–S7 and Tables S1–S3 [file mmc1.pdf]

Cell Reports, Volume *44*

Supplemental information

Lack of context modulation in human single neuron responses in the medial temporal lobe

Hernan G. Rey, Theofanis I. Panagiotaropoulos, Lorenzo Gutierrez, Fernando J. Chaure, Alejandro Nasimbera, Santiago Cordisco, Fabian Nishida, Antonio Valentin, Gonzalo Alarcon, Mark P. Richardson, Silvia Kochen, and Rodrigo Quian Quiroga

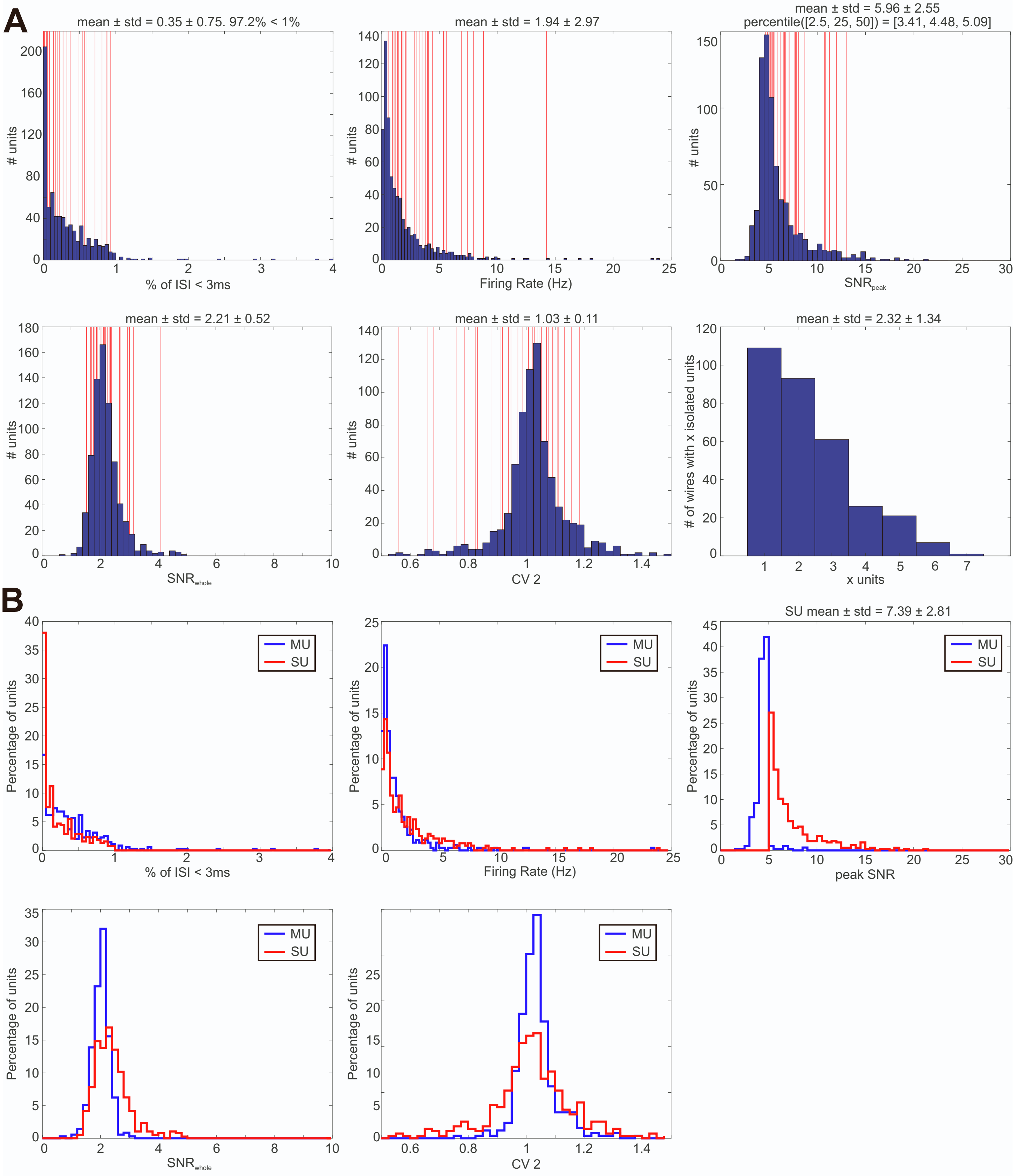

Figure S1. Spike sorting quality metrics. (A) For all the units isolated in the dataset we computed the percentage of ISI that were less than 3ms, the firing rate (over the whole recording session), the SNR_{peak} (using the peak amplitude of the mean waveform), the SNR_{whole} (considering the SNR across the whole waveform), the modified coefficient of variation, and the distribution of isolated units per wire (see Methods for all the specific definitions of the metrics). Vertical lines denote the individual values for the 33 units that showed significant identity responses during the encoding phase of the paradigm. (B), Same as before but the distributions were calculated separately for single (SU) and multi (MU) unit populations.

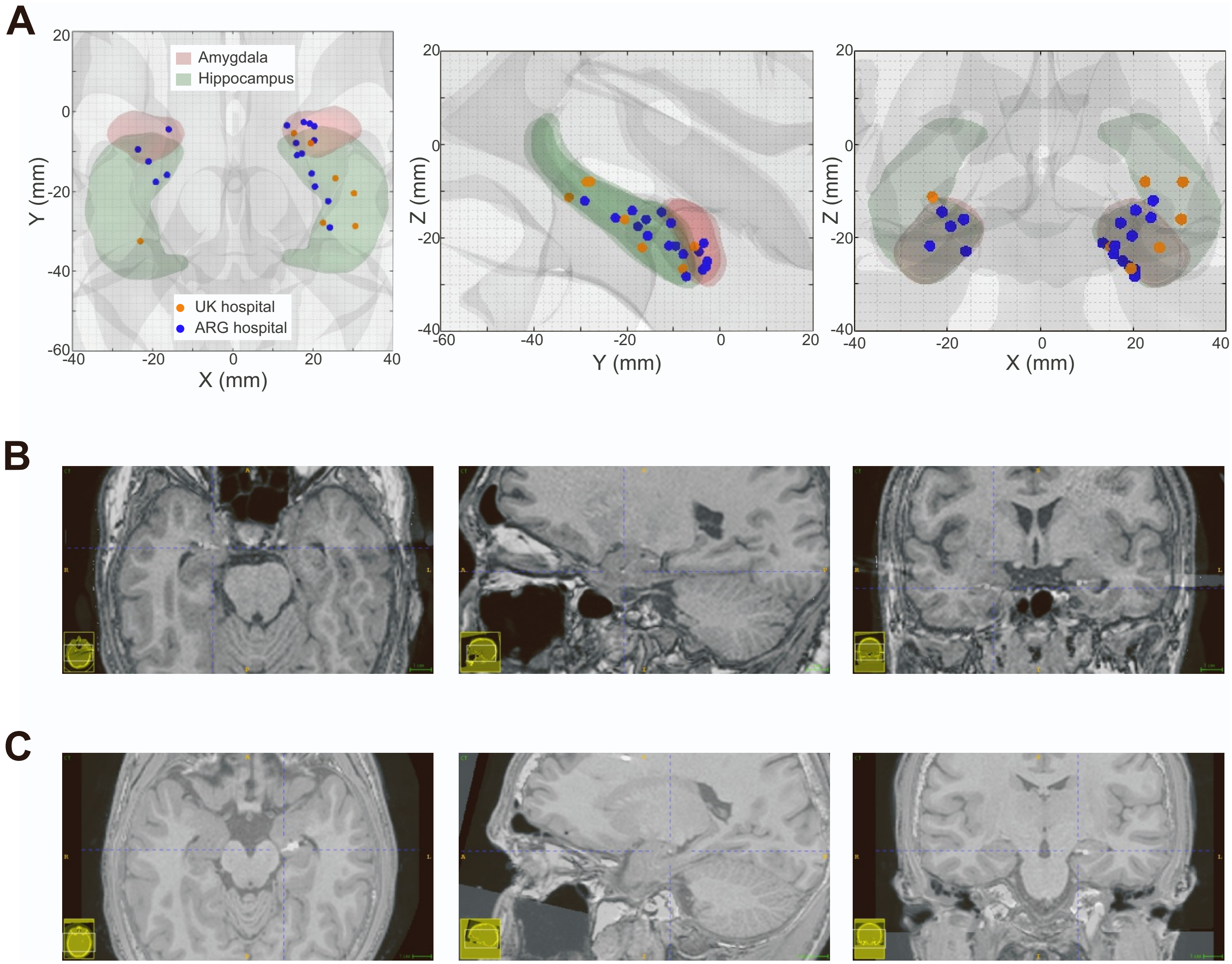

Figure S2. Electrode implantation. (A), microelectrode localization for all the probes included in the current dataset (color coded according to the hospitals where they were implanted). Hippocampus and amygdala are shown in green and red, with 15 and 9 probes reaching the respective structures. From left to right, each panel shows axial, sagittal, and coronal views. (B), CT-MRI coregistration showing an example of a probe reaching the right amygdala. Note the artefact in the CT generated by the microwire bundle. (C), Same as (B), but for a probe reaching the left hippocampus.

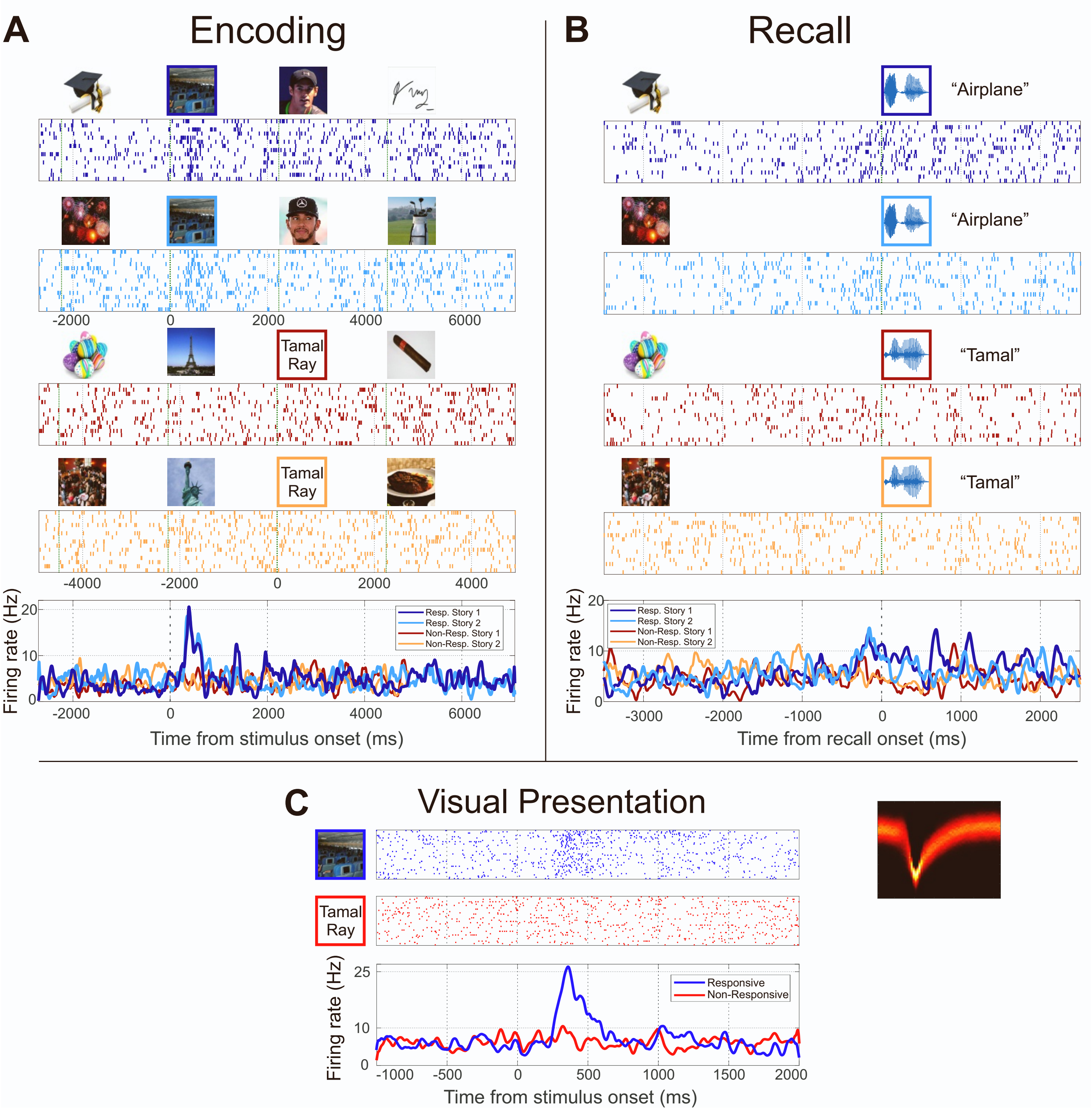

Figure S3. Exemplary neuron responding to a place. Hippocampal neuron responsive to a picture of an *airplane* cabin, but not to *Tamal* (a contestant in the TV show *British Bake Off*), during encoding (A), recall (B), and visual presentation (VP) (C). A density plot of the waveforms associated with the putative single neuron is also shown. The identity effect in the ANOVA test was significant during encoding ($p \sim 10^{-8}$, total df = 59), recall ($p \sim 10^{-5}$, total df = 58), and VP ($p \sim 10^{-5}$, total df = 59). There was no significant effect for the story both during encoding (p = 0.45) and recall (p = 0.33). Copyright notes: Picture of Andy Murray was cropped from “2015 Australian Open - Andy Murray 6” by Brendan Dennis, licensed under CC BY-SA 4.0. Picture of Lewis Hamilton was cropped from “Lewis Hamilton 2016 Malaysia 2” by Morio, licensed under CC BY-SA 4.0.

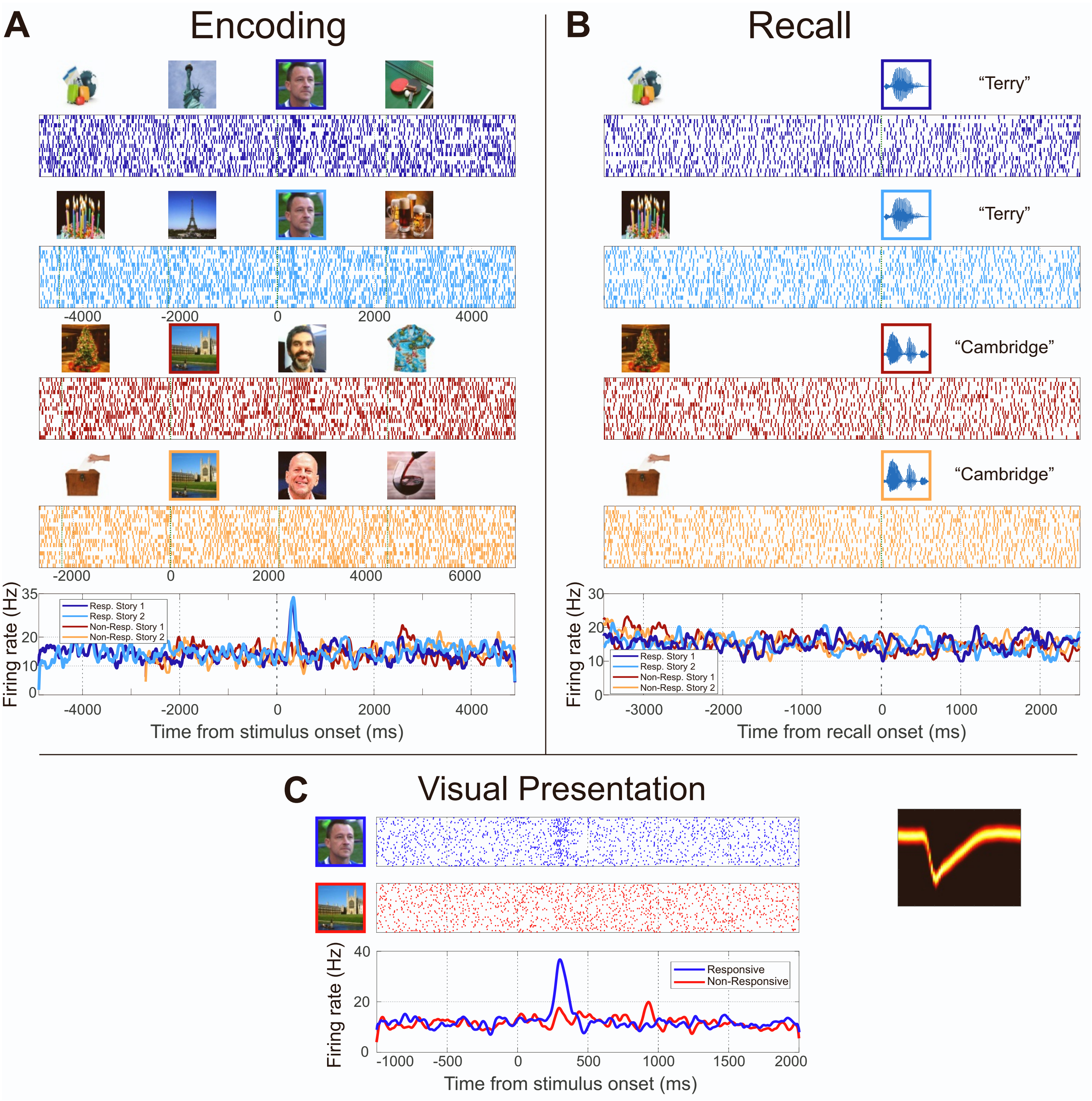

Figure S4. **Exemplary neuron that responded during encoding and VP, but not during recall**. Hippocampal neuron that responded to *John Terry* (former captain of Chelsea FC), but not to the *King's College Chapel at the University of Cambridge*, during encoding ($p \sim 10^{-6}$, total df = 59) (A) and VP ($p \sim 10^{-3}$, total df = 79) (C), but not during recall ($p = 0.23$, total df = 59) (B). There was no significant effect for the story both during encoding ($p = 0.1$) and recall ($p = 0.18$). A density plot of the waveforms associated with the putative single neuron is also shown. Copyright notes: Picture of John Terry was cropped from "Chelsea 5 Sunderland 1 (34821048206)" by @cfcunofficial, licensed under CC BY-SA 2.0. Picture of Bruce Willis was cropped from "Bruce Willis (4840565824)" by Gage Skidmore, licensed under CC BY-SA 2.0. Picture of Dr Antonio Valentin (co-author of the paper) is a self-portrait.

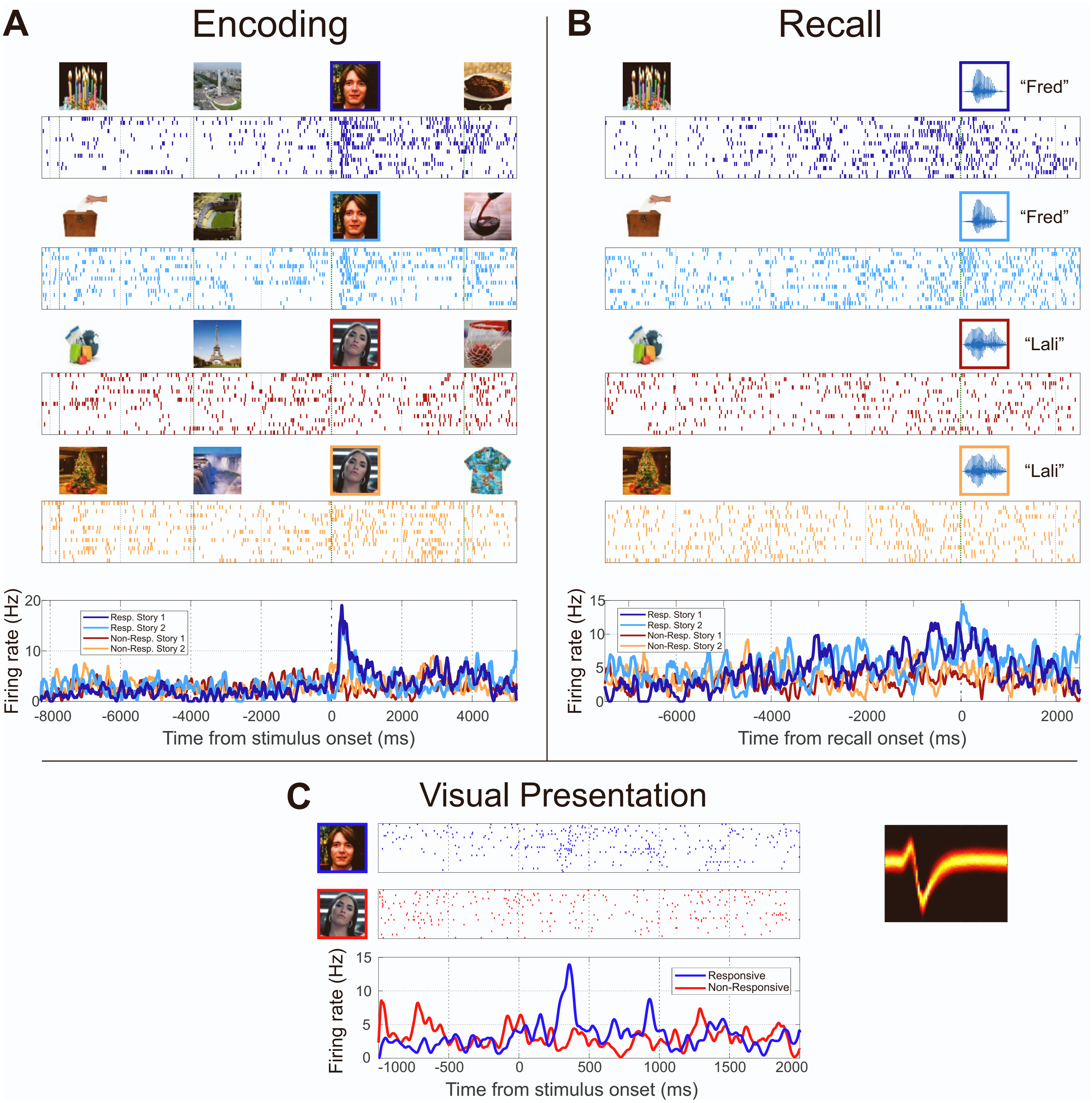

Figure S5. **Exemplary neuron that responded during encoding, VP and recall**. Hippocampal neuron with a response to *Fred Weasley* (a character from the movie *Harry Potter*), but not to *Lali Esposito* (a singer from Argentina), during encoding ($p \sim 10^{-10}$, total df = 59) (A), recall ($p \sim 10^{-8}$, total df = 58) (B), and VP ($p \sim 10^{-3}$, total df = 59) (C). There was no significant effect for the story both during encoding ($p = 0.07$) and recall ($p = 0.014$). A density plot of the waveforms associated with the putative single neuron is also shown.

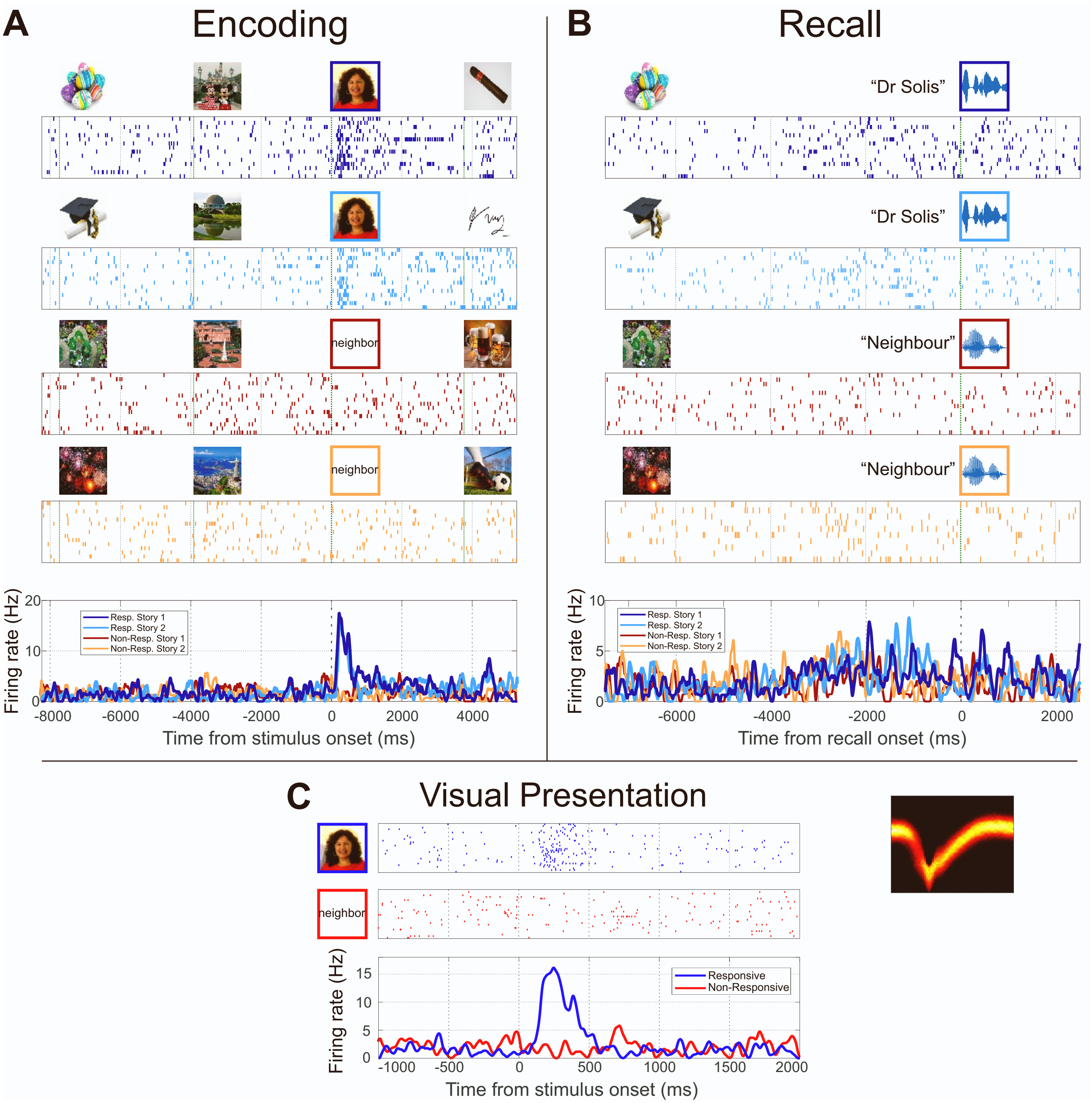

Figure S6.Exemplary neuron from the amygdala that responded during encoding, VP and recall. The neuron shows a response to *Dr Patricia Solis* (a neuropsychologists that was part of the clinical team treating the patient during their hospital stay), but not to the *picture of the patient's neighbor*, during encoding (p ~10^{-12}, total df = 59) (A), recall (p ~10^{-3}, total df = 53) (B), and VP (p ~10^{-10}, total df = 59) (C). There was no significant effect for the story both during encoding (p = 0.14) and recall (p = 0.57). A density plot of the waveforms associated with the putative single neuron is also shown. Picture of Dr Patricia Solis (who was part of the clinical team) is a self-portrait.

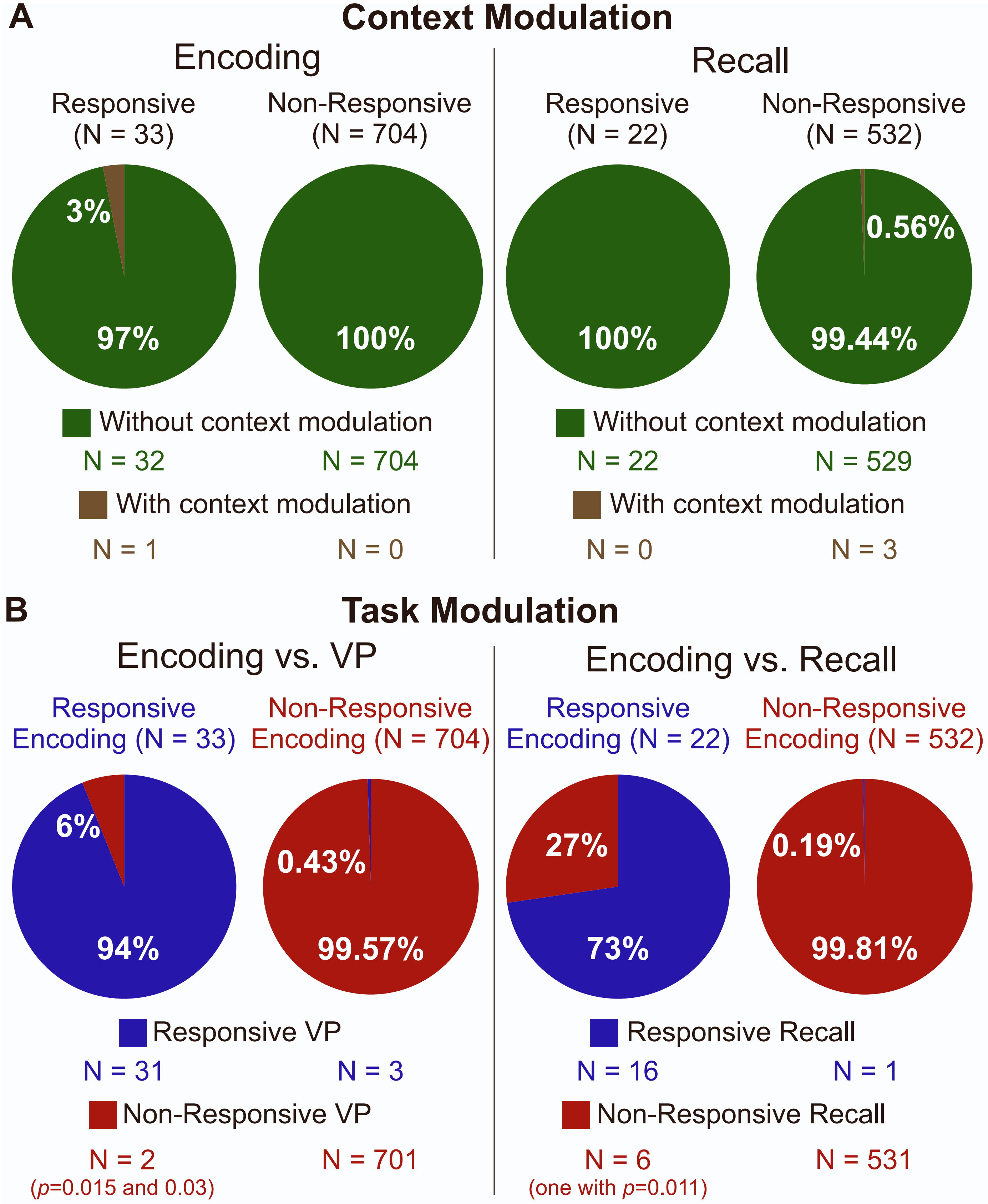

Figure S7. Number of neurons showing context and task modulations based on the ANOVA results. (A), Responsive (left) and non-responsive neurons (right) showing context modulation during encoding and recall. (B), Neurons compared across different tasks. Most of the neurons that responded significantly to a given identity during encoding, also responded to the same identity during the visual presentation task (with two neurons being close to the significance threshold). A large percentage of those neurons also responded during recall (with the activity being driven by an internal process without external stimulation). The vast majority of neurons not eliciting responses during encoding were also not responsive during VP and recall.

Session number	1	2	3	4	5	6	7	8	9	10	11	12	13	14	15	16	17	18	19	20	21	Total
# neurons in Hippocampus	32	42	36	38	14	25	33	10	-	56	37	-	-	-	-	11	10	11	28	27	21	431
# neurons in Amygdala	20	19	13	9	5	-	37	5	17	11	26	23	25	18	18	-	-	-	23	19	18	306
# resp. during encoding in Hippocampus	1	1	2	2	2	1	2	2	0	1	1	0	0	0	0	1	1	2	0	0	0	19
# resp. during encoding in Amygdala	1	0	1	0	0	0	0	0	2	0	0	1	1	1	2	0	0	0	3	1	1	14

Patient id	1	2	3	4	5	6	7	8	9	mean ± std
# resp. per session	2	1.5	2	2	1	1	1.25	1.33	1.66	1.53 ± 0.41

Table S1. Number of units recorded on each area (amygdala and hippocampus) per experimental session. We also show the distribution across experimental sessions of all the 33 neurons eliciting a response during the encoding phase. Sessions 1-4 correspond to patient 1; sessions 5-6 correspond to patient 2 (both of them from the UK); sessions 7-8 correspond to patient 3; sessions 9, 10, and 11 correspond to patient 4, 5 and 6, respectively; sessions 12-15 correspond to patient 7; sessions 16-18 correspond to patient 8; sessions 19-21 correspond to patient 9. To better visualize the contribution of each patient to the dataset, we quantified the average number of observed responsive neurons per session.

	$p < 0.01$	$p < 0.05$
ANOVA test with responsive neurons during encoding (N = 33)	1 [a]	2
ANOVA test with non-responsive neurons during encoding (N = 704)	0	3
ANOVA test with responsive neurons during recall (N = 22)	0	3 [b]
ANOVA test with non-responsive neurons during recall (N = 532)	3	5
Surrogate test on strength with responsive neurons during encoding (N = 33)	0	1
Surrogate test on latency with responsive neurons during encoding (N = 33)	0	0
Surrogate test on strength with non-responsive neurons during encoding (N = 2 x 704 = 1,408)	2	6
Surrogate test on strength with responsive neurons during recall (N = 22)	0	0
Surrogate test on strength with non-responsive neurons during recall (N = 2 x 532 = 1,064)	2	14
Decoding during encoding sessions (N = 21)	0	0
Decoding during recall sessions (N = 14)	0	1

Table S2. Number of cases showing significant effects for context/story using different tests and significant levels. The surrogate tests on the non-responsive set of neurons was applied to each identity separately, leading to a number of cases that was twice the number of neurons. For decoding results, each case was an experimental session. [a] This neuron is shown in **Fig. 2C**. [b] One of these neurons is shown in **Figure S5**.

	$\alpha = \beta = 0.01$	$\alpha = \beta = 0.05$
Statistically equivalent tests on responsive neurons during encoding	32 out of 32 (100%)	31 out of 31 (100%)
Statistically equivalent tests on non-responsive neurons during encoding	1382 out of 1408 (98%)	1333 out of 1402 (95%)
Statistically equivalent tests on responsive neurons during recall	22 out of 22 (100%)	18 out of 19 (95%)
Statistically equivalent tests on non-responsive neurons during recall	1033 out of 1058 (98%)	1003 out of 1054 (95%)

Table S3. Number of cases showing statistically equivalent differences in strength for the different pairs of stories from a given identity. For each pair of stories from a given identity in a neuron that led to non-significant results using the ANOVA test, we evaluated if their difference in strength was statistically equivalent using the TOST approach (see Methods). The test outcome was “statistically equivalent” when the composite hypotheses were rejected at level α.
