# Supplementary material for: Lack of context modulation in human single neuron responses in the medial temporal lobe
Source: Cell Rep. 2025 Jan 15;44(1):115218. doi: 10.1016/j.celrep.2024.115218 (PMC11781864; doi:10.1016/j.celrep.2024.115218)
Supplement: Document S2. Article plus supplemental information [file mmc2.pdf]

Cell Reports

Article

Lack of context modulation in human single neuron responses in the medial temporal lobe

Graphical abstract

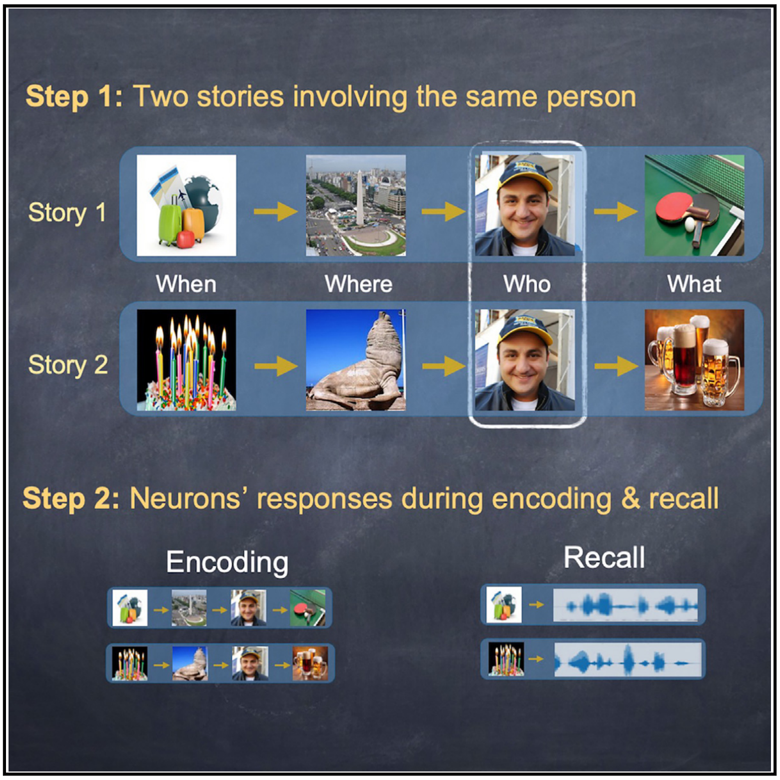

Authors

Hernan G. Rey,
Theofanis I. Panagiotaropoulos,
Lorenzo Gutierrez, ...,
Mark P. Richardson, Silvia Kochen,
Rodrigo Quian Quiroga

Correspondence

rqqg1@le.ac.uk

In brief

Rey et al. use a naturalistic memory task, in which subjects learn and then recall two different stories involving the same person in different contexts, while recording the activity of individual hippocampal neurons. They report similar single-neuron responses to the same identity in different contexts and tasks.

Highlights

- In a naturalistic memory task, we show that hippocampal neurons are not modulated by context
- We observe similar single-neuron responses to the same concepts in different tasks
- Neurons do not show conjunctive coding (i.e., responding only in specific contexts or tasks)
- This context-invariant coding could be a key, unique feature of human intelligence

Rey et al., 2025, Cell Reports *44*, 115218
January 28, 2025 © 2025 The Authors. Published by Elsevier Inc.
https://doi.org/10.1016/j.celrep.2024.115218

Cell Reports

CellPress
OPEN ACCESS

Article

Lack of context modulation in human single neuron responses in the medial temporal lobe

Hernan G. Rey,[1,2] Theofanis I. Panagiotaropoulos,[1,3,12,13] Lorenzo Gutierrez,[1,11] Fernando J. Chaure,[1,4] Alejandro Nasimbera,[5] Santiago Cordisco,[5,11] Fabian Nishida,[5] Antonio Valentin,[6] Gonzalo Alarcon,[7] Mark P. Richardson,[6] Silvia Kochen,[5,8] and Rodrigo Quian Quiroga[1,9,10,11,14,*]

[1]Centre for Systems Neuroscience, University of Leicester, Leicester, UK
[2]Departments of Neurosurgery, Biomedical Engineering, and Pharmacology and Toxicology, Medical College of Wisconsin, Milwaukee, WI 53226, USA
[3]Cognitive Neuroimaging Unit, INSERM, Universite Paris-Sud, Universite Paris-Saclay, Paris, France
[4]Institute of Biomedical Engineering, University of Buenos Aires, Buenos Aires, Argentina
[5]ENyS, CEMET, Av. Calchaquí 5401, Buenos Aires 1888, Argentina
[6]Department of Basic and Clinical Neuroscience, Institute of Psychiatry Psychology and Neuroscience, King's College London, London, UK
[7]Department of Clinical Neurophysiology. Royal Manchester Children's Hospital, Manchester, UK
[8]Epilepsy Centre, El Cruce Hospital, Buenos Aires, Argentina
[9]Hospital Del Mar Medical Research Institute (IMIM), Barcelona, Spain
[10]Institució Catalana de Recerca I Estudis Avançats (ICREA), Barcelona, Spain
[11]Ruijin Hospital, Shanghai Jiao Tong University School of Medicine, Shanghai, China
[12]Department of Psychology, National and Kapodistrian University of Athens, 15784 Athens, Greece
[13]Centre for Basic Research, Biomedical Research Foundation of the Academy of Athens (BRFAA), Athens, Greece
[14]Lead contact
*Correspondence: rqqg1@le.ac.uk
https://doi.org/10.1016/j.celrep.2024.115218

SUMMARY

In subjects implanted with intracranial electrodes, we use two different stories involving the same person (or place) to evaluate whether and to what extent context modulates human single-neuron responses. Nearly all neurons (97% during encoding and 100% during recall) initially responding to a person/place do not modulate their response with context. Likewise, nearly none (<1%) of the initially non-responsive neurons show conjunctive coding, responding to particular persons/places in a particular context during the tasks. In line with these findings, taking all neurons together it is possible to decode the person/place being depicted in each story, but not the particular story. Moreover, the neurons show consistent results across encoding and recall of the stories and during passive viewing of pictures. These results suggest a context invariant, non-conjunctive coding of memories at the single-neuron level in the human hippocampus and amygdala, in contrast to what has been described in other species.

INTRODUCTION

A plethora of studies in rodents and monkeys have consistently shown that hippocampal neurons tend to change their responses when varying the environment or the task performed by the animals, thus providing the neuronal machinery to differentiate memories in different contexts.[1–13] Consistent with these results and with the well-established role of the hippocampus in memory,[14,15] non-invasive studies in humans (mainly fMRI) have also demonstrated clear context modulations in hippocampal responses.[16–29] However, there is still no direct evidence in humans of how and to what extent single neurons may modify their response patterns when varying the context to differentiate memories. To address this issue, here, we use the unique opportunity to record multiple single neurons in the medial temporal lobe (MTL) of patients implanted with intracranial electrodes for clinical reasons[30,31] and studied for the first time whether the firing of these neurons is modulated when asking subjects to learn and then recall stories involving different contexts. In particular, we ask the following questions: if a neuron fires to a particular person, how will it respond in two different stories involving the same person? Conversely, will initially non-responsive neurons start firing to a conjunction of features, such as a person in one (but not the other) story, to discriminate between them? Surprisingly, and in contrast to findings in rodent and monkey hippocampuses,[1–3,8] we found no evidence of conjunctive coding, and in the vast majority of cases, the neurons did not change their activity when changing the context and the task performed by the subjects.

RESULTS

Single-neuron responses during visual presentation, encoding, and recall

To evaluate how neuronal responses are modulated (or not) by context in the human MTL, we recorded the activity of 737 units

© 2025 The Authors. Published by Elsevier Inc.
This is an open access article under the CC BY license (http://creativecommons.org/licenses/by/4.0/).

in the hippocampus and amygdala (384 single units and 353 multiunits; see STAR Methods; Figure S1; Table S1), during 21 experimental sessions in 9 patients with drug-resistant epilepsy who were candidates for surgical treatment and were implanted with intracranial electrodes[30,31] (see STAR Methods; Figure S2). In each experimental session, after selecting two identities (e.g., two famous actors) for which we had neuronal responses in different neurons (as determined from previous screening sessions; see STAR Methods), the subjects performed a memory task in which they learned and then recalled four simple stories, two stories involving different contexts for each identity. We stress that (1) the criterion for selecting the identities was only that they elicited a response in any of the recorded neurons (i.e., without examining any further the characteristics of the responses, such as visual invariance) and (2) we analyzed all the recorded neurons (first the responsive ones, then the non-responsive ones, and finally all the recorded neurons together using a decoding approach), and the identities eliciting responses on a given neuron could be considered non-responsive (i.e., random identities) for the other neurons. The four stories had the same structure, with a temporal context (when), followed by a spatial context (where), then a person (who), and finally an action (what) (Figure 1A; see STAR Methods), and were shown in pseudorandom order during the encoding phase. The presentation of the stories was followed by a recall phase, where the subjects had to verbally recall each of the four stories in pseudorandom order, after being cued by the picture representing the corresponding temporal context (STAR Methods). A recall trial was considered correct if the subject correctly retrieved the place, person, and action corresponding to the story associated with the temporal context cue. The encoding-recall blocks were repeated between 10 and 15 times. Figure 1B shows the behavioral performance of the subjects (see STAR Methods), where it can be observed that they were able to learn the stories after a few trials. Furthermore, to verify that the selected identities still elicited a response in the corresponding neurons (as recording conditions may change compared to the previous screening sessions), we also ran a simple visual presentation (VP) task, where the subjects saw all the stimuli used during the stories between 15 and 30 times each and in pseudorandom order (STAR Methods).

A total of 33 of the recorded units had a significantly larger response to one of the identities during the encoding phase (two-way analysis of variance [ANOVA], factor identity, $p < 0.01$; see STAR Methods; in five of these cases the response was to a place, and we created the two contexts by having a different person in two stories featuring the same place; see Figure S3). Most of these neurons (31 out of 33, 94%) also had a significant response to the preferred stimulus during the VP task (one-way ANOVA, $p < 0.01$; see STAR Methods), and in the other two cases, the responses were close to significant, with $p = 0.015$ and 0.03, respectively. This suggests that responses were task independent, with the units responding to the same identities in a visual perception task and a memory task (see below).

Figure 2 shows an exemplary neuron from the left hippocampus that responded to Diego Topa (an Argentinian television host) but not to the actor Jackie Chan. During the VP task, the neuron exhibited a clear response to Diego Topa, increasing its firing to about 30 Hz, from a baseline of ~5 Hz. During the encoding phase, the neuron showed a similar response, increasing its firing rate to about 25 Hz upon the presentation of Diego Topa, regardless of the specific story (involving different contexts), while it did not show a change from baseline for Jackie Chan. The two-way ANOVA confirmed the effect of identity ($p \sim 10^{-17}$), whereas a modulation of the response of the neuron by the different stories was not significant ($p = 0.84$), emphasizing the lack of difference across contexts for the stories involving Diego Topa. In fact, of all 33 neurons showing an identity effect, only one neuron (3%), depicted in Figure 2C, showed a significant modulation of the activity by the story ($p = 0.003$, two-way ANOVA, factor context; see STAR Methods). To further quantify these results, we also performed independent permutation tests on the strength and latency of the responses (STAR Methods), evaluating whether there was a significant difference between the stories associated with a responsive identity. None of the 33 neurons showed significant differences in strength or latency (the one in Figure 2C was close to significant for strength but not for latency). Results were qualitatively similar when considering different criteria of statistical significance (Table S2).

We then analyzed the neuronal responses during the recall phase, which we were able to record in 14 of the 21 sessions (see STAR Methods); in other words, from the 33 responsive neurons observed during encoding, we could analyze 22 of them during recall. We found that 16 of them showed a significant difference for stimulus identity (two-way ANOVA, $p < 0.01$, one of the other six cases had $p = 0.011$; Figure S4 shows one of the neurons that exhibited responses during encoding but not during recall). Figure 3 depicts the same neurons as Figure 2 but during the recall phase. We first observe that recall responses do not exhibit a clear onset as in encoding and VP. This is because the activity is driven by an internal process and we aligned the trials to the beginning of the vocalization of the identity of interest, as in previous works.[32,33] Moreover, we observe that the responses show an increased firing rate prior to the recall of the preferred identities, which is similar for both stories. In fact, none of the 22 neurons showed a significant difference for the stories (two-way ANOVA, factor context, $p < 0.01$), and none of them reached significance in the permutation tests, comparing strength differences across stories for a given responsive identity (given the absence of a well-defined response onset, we did not test latency effects during recall). As before, results were qualitatively the same when considering different statistical criteria (Table S2).

Single neurons maintain selectivity across tasks

Figure 4A shows the normalized instantaneous firing rate of the 33 neurons that had a significant identity effect during the encoding phase; the grand average results are presented in Figure 4B. The response profile is very similar across the two stories involving the identity eliciting the responses of the neurons and, as expected, much larger than the ones to the stories involving the identity not eliciting responses, thus emphasizing the finding of differential responses for the different identities but not for the different stories. Moreover, the response profile during the encoding phase is very similar to the one obtained during VP, showing that the neuronal responses tend to be maintained

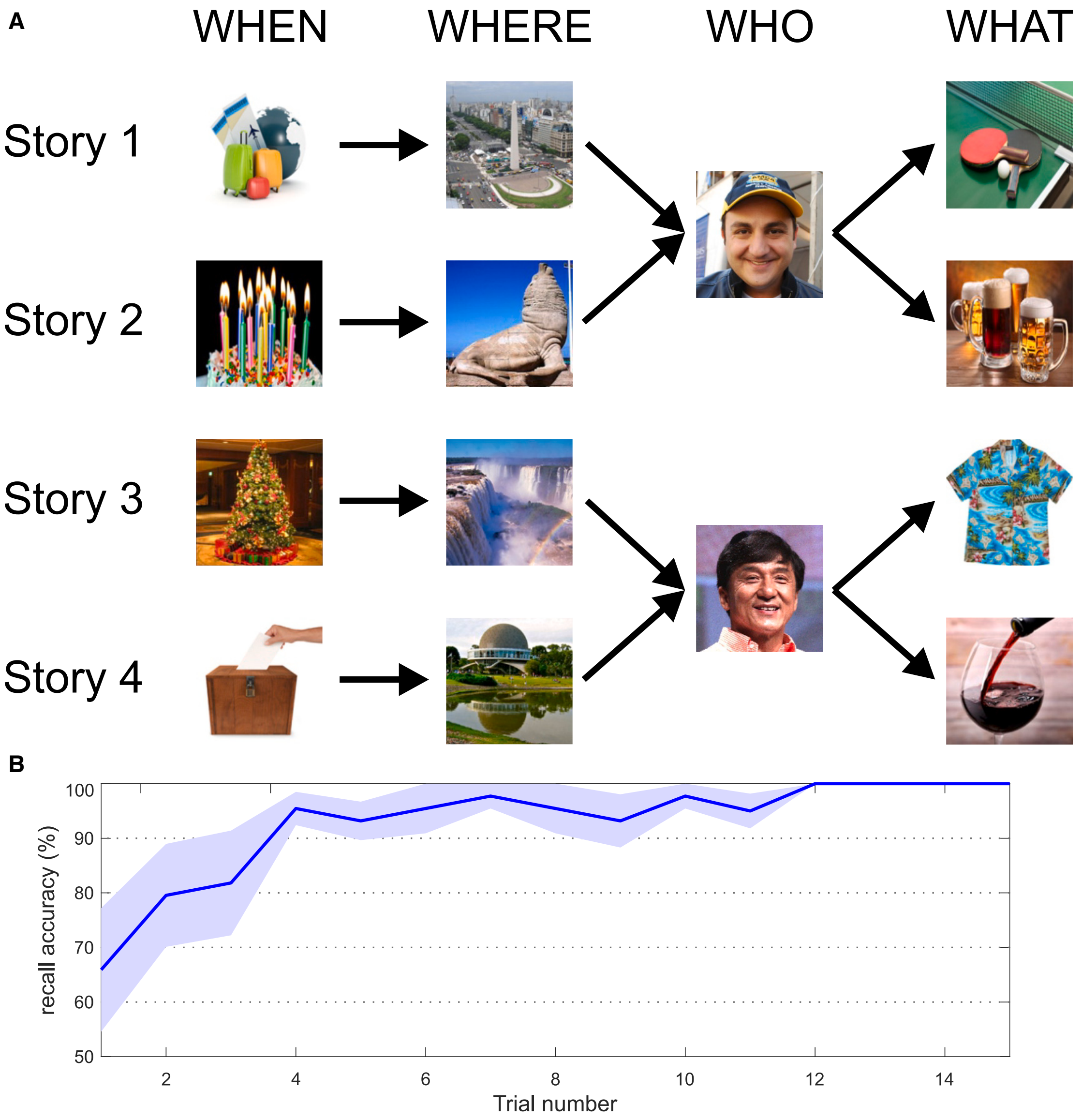

Figure 1. Behavioral task

(A) Structure of the experimental paradigm. Two stimuli were selected for the task (in this example, Jackie Chan and Diego Topa), each of which was embedded in two different stories, reflecting two simple episodic memories in very different contexts. All four stories had a similar structure: "when," "where," "who," and "what." First, there was a temporal context (e.g., "Last year, a strange event took place on Christmas"), which was then followed by a place/spatial context (e.g., "I was traveling around, and I ended up visiting the Iguazu Waterfalls") and then a person (e.g., "Then, out of nowhere, I spotted Jackie Chan"), and finally, there was a conclusion (e.g., "After chatting for a while, he told me he liked my colorful shirt"). On each trial, the four stories were presented in pseudorandom order. Subjects were asked to remember the stories presented during these encoding trials, which were followed by the recall trials, in which the subjects saw the picture of the temporal context ("when") presented for 1 s and had 12 s to verbally recall the associated story (with the trial considered correct if the subject was able to retrieve the corresponding "where," "who," and "what" components). This trial structure (encoding-recall of the four stories) was repeated between 10 and 15 times.

(B) Subjects managed to correctly learn the different stories after a few trials, as measured by the recall performance (percentage of correctly recalled stories; mean ± SEM).

The photograph of Diego Topa was modified from a photograph by Mariaesterramos, licensed under CC-BY-SA-3.0. The photograph of Jackie Chan was modified from a photograph by Gage Skidmore, licensed under CC-BY-SA-3.0.

across different tasks. The response patterns are different during cued recall because in this case there is not a well-defined stimulus onset; in other words, the responses are triggered by internal processes and are therefore smeared in time, starting seconds before naming the identity, whereas during VP and encoding the responses are evoked by the picture presentation and have a consistent and marked response onset a couple hundred milliseconds after stimulus presentation. However, as in the encoding

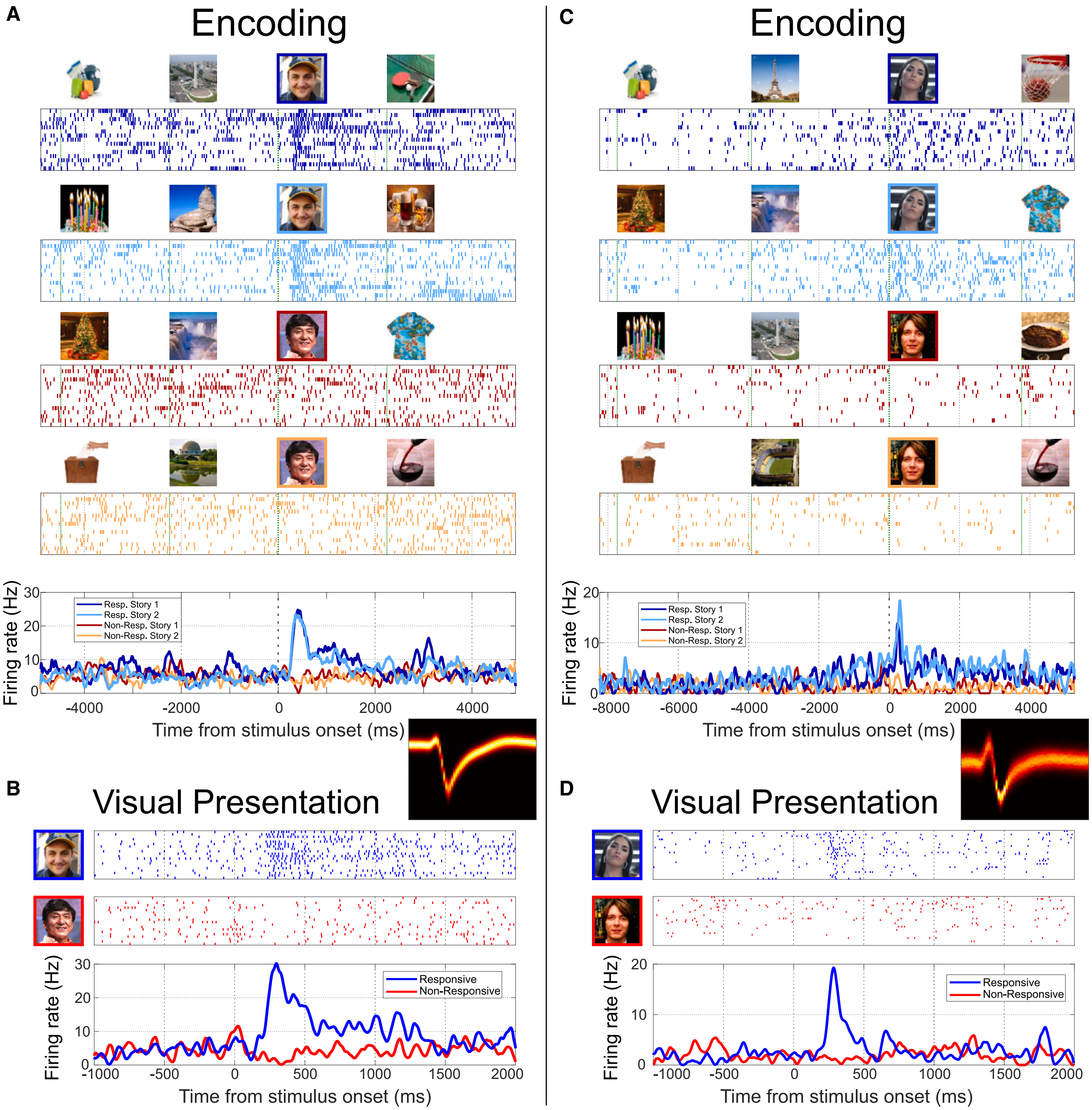

Figure 2. Exemplary responses during the encoding and visual presentation phases

(A and B) Responses from a unit in the left anterior hippocampus during encoding and visual presentation (VP). In each case, the raster plots (first trial on top) and instantaneous firing rates are shown. Time zero corresponds to the onset of the relevant stimulus (pictures highlighted with a colored square). The spike density plot of the corresponding neuron is also shown. The neuron shows a strong response to Diego Topa during both encoding and VP, without differences between the two stories involving him. No activity change is noticeable in response to the picture/stories of Jackie Chan. Two-way ANOVA encoding (total degrees of freedom [df] = 59): p(identity) $\sim 10^{-17}$, p(context) = 0.84. One-way ANOVA VP (total df = 39): $p \sim 10^{-12}$.

(C and D) Another example from a neuron in the left anterior hippocampus of a different patient. During VP, the neuron exhibited a strong response to Lali Esposito (a singer from Argentina) but not to Fred Weasley (a character from the *Harry Potter* movies). In the encoding phase, there was a strong effect on identity (responding to Lali and not to Fred) but also a significant effect for story. Two-way ANOVA encoding (total df = 59): p(identity) $\sim 10^{-15}$, p(context) = 0.003. One-way ANOVA VP (total df = 59): $p \sim 10^{-5}$

The photograph of Lali Esposito was modified from a photograph by Fernando Radl, licensed under CC-BY-SA-3.0. The photograph of Fred Weasley was modified from a photograph by Niccolo Caranti, licensed under CC-BY-SA-3.0.

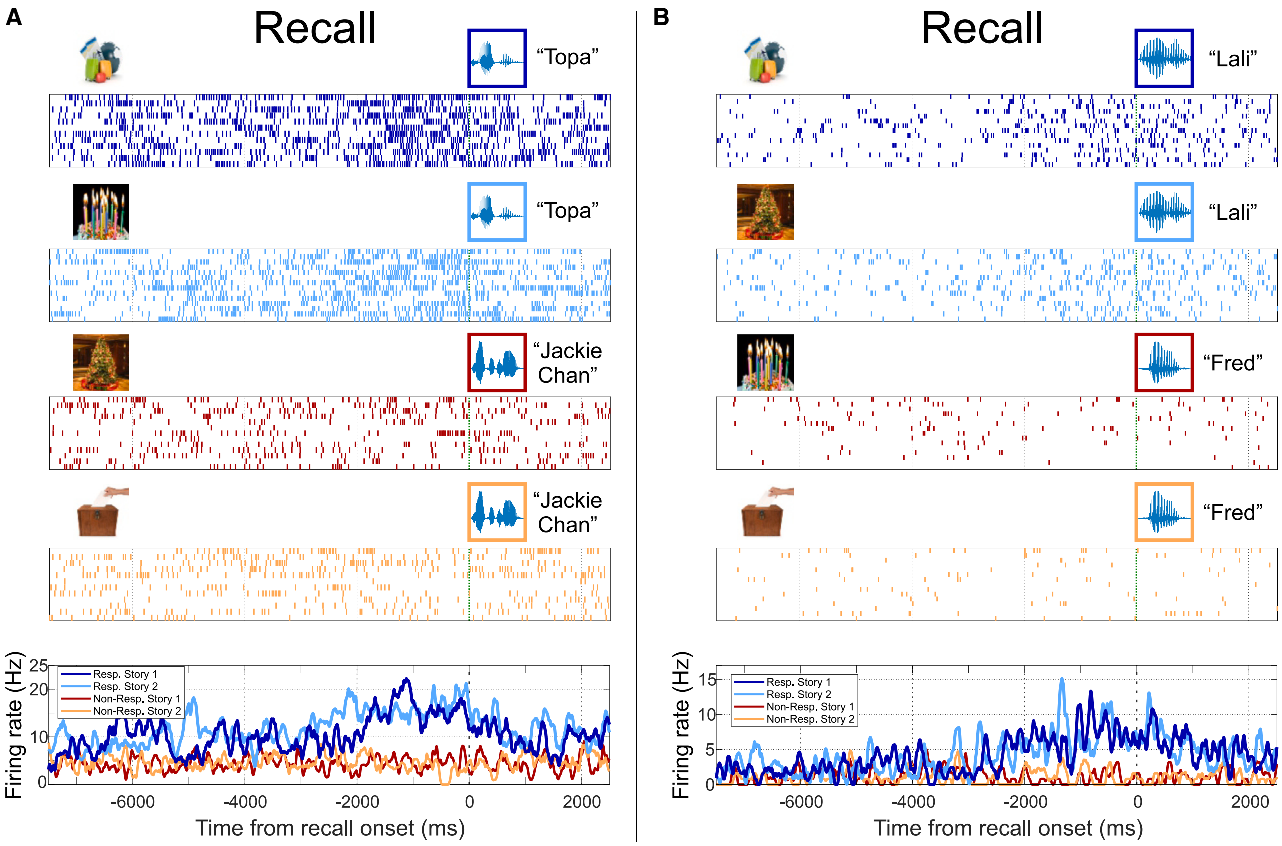

Figure 3. Exemplary responses during the recall phase

Responses from the same neurons presented in Figure 2 but now during the recall phase. Time zero was defined as the moment where the subject started vocalizing the name of the relevant person (highlighted with a colored square) during the recall of the story. Neurons responded similarly to both stories involving (A) Diego Topa (two-way ANOVA [total df = 50]: p[identity] $\sim 10^{-15}$, p[context] = 0.07) and (B) Lali (two-way ANOVA [total df = 58]: p[identity] $\sim 10^{-22}$, p[context] = 0.33).

and VP phases, there is a larger activation for the stimuli eliciting responses (in contrast to the non-responsive ones) and no clear differences between the different stories. Although the responses during recall are clearly different from those during VP and encoding, these tasks activate the same neurons. In particular, there is a strong correspondence between the neurons activated during encoding and recall (73% for the responsive neurons and nearly 100% for the non-responsive ones; see Figure S7), as well as during VP and encoding (94% for the responsive neurons and nearly 100% for the non-responsive ones).

Figure 4C depicts the strength of the responses during encoding and recall (left and right plots, respectively) for different stories (see STAR Methods). Both for encoding and recall we observe a large variability of the responses of the different units, but similar responses to the two stories (i.e., the points placed roughly along the diagonal). In fact, the activity across the two stories for the response-eliciting identities were significantly correlated (Pearson encoding: $r = 0.65$, $p \sim 10^{-5}$; recall: $r = 0.89$, $p \sim 10^{-6}$), in line with the previous observation that for most neurons, the responses to both stories were statistically the same during encoding and recall. Figure 4D shows the responses in the different tasks. We found large and significant correlations when comparing the responses across tasks (Pearson encoding vs. VP: $r = 0.60$, $p \sim 10^{-7}$; recall vs. VP: $r = 0.69$, $p \sim 10^{-7}$; encoding vs. recall: $r = 0.60$, $p \sim 10^{-4}$). Corroborating the previous results, the individual neurons exhibit similar activity not only across the two stories associated with the response-eliciting identity but also across different tasks (passive VP, memory encoding, and memory recall).

Initially non-responsive neurons do not show conjunctive coding

Next, we analyzed the remaining 704 neurons that did not elicit a significant effect for identity during the encoding phase. None of them showed a significantly different response for the two stories (two-way ANOVA, $p < 0.01$). Again, qualitatively similar results were obtained with the permutation tests and when using different statistical criteria (Table S2). Considering the same group of 704 neurons during the VP task, we observed significant responses in only three of them (0.3%), which is less than what is expected by chance. In the recall phase, we recorded 532 out of the 704 neurons that did not respond in the encoding phase (see STAR Methods). Only one of the 532 neurons (0.15%) showed a significant difference for identity and three (0.45%) showed a

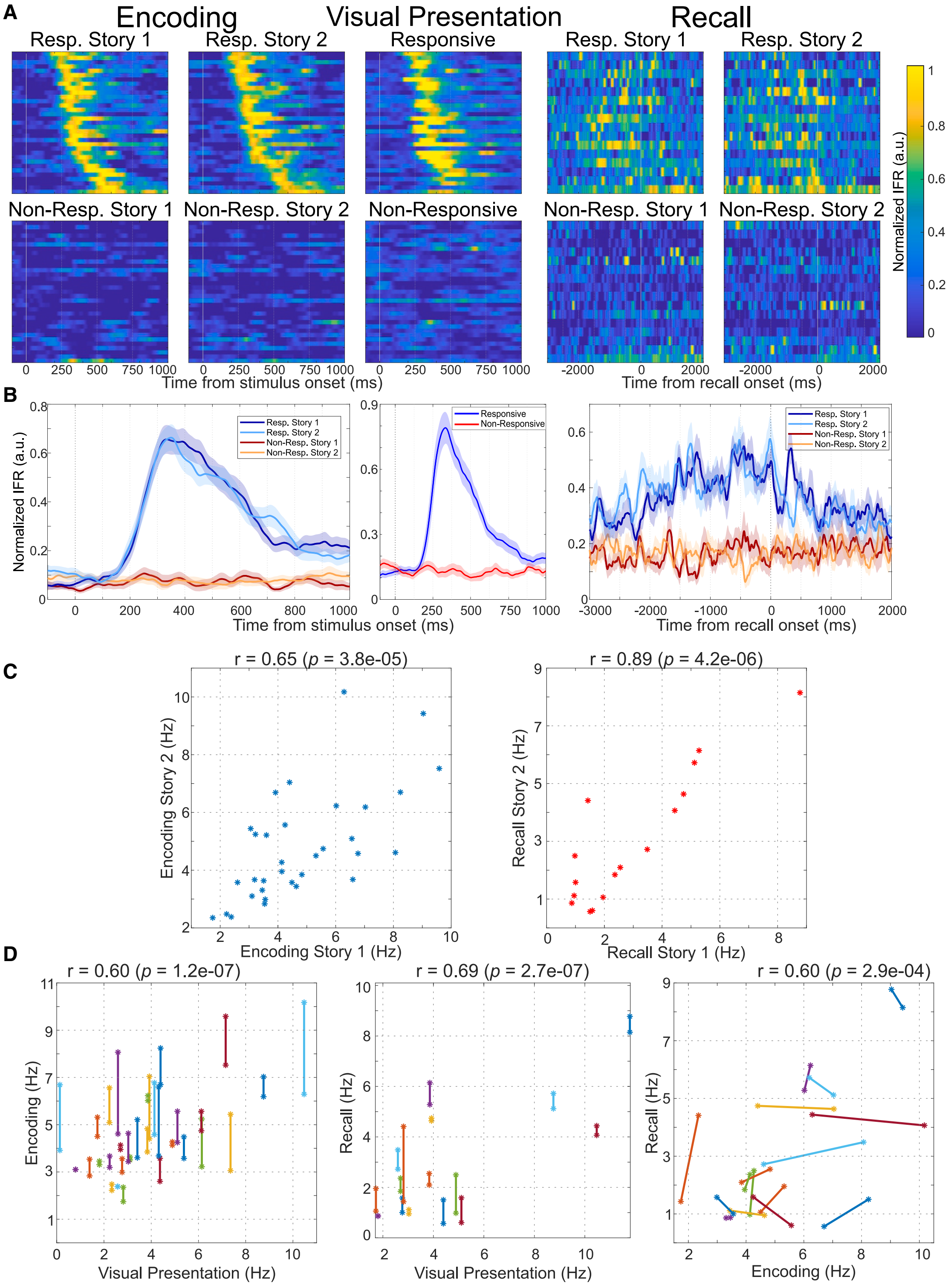

Figure 4. Firing of responsive neurons in the different tasks

(A) Normalized instantaneous firing rate for all responsive neurons (33, one per row) during the encoding, VP (matching the 33 neurons in encoding), and recall phases (for the latter, we included only the 16 that had a significant identity effect). All neurons were sorted by the average response latency of the stories eliciting a response in the encoding phase. The population profile for the responsive identities is very similar for both stories during the encoding phase, but also in

(legend continued on next page)

significant difference for story (two-way ANOVA, $p < 0.01$), which is less than what is expected by chance. During recall, qualitatively similar results were also obtained with the permutation tests comparing the strength of the responses and with different statistical criteria (Table S2). A summary of the number of neurons with context and task modulations in the different conditions is shown in Figure S7, which shows that similar responses were obtained across tasks, and that the vast majority of both the responsive and non-responsive neurons showed no context modulations.

Further quantifications that single neuron responses do not show context modulations

Given that our main result is the absence of evidence for significant differences, we used a statistical equivalence test, the two one-sided tests procedure (see STAR Methods), to determine the strength of evidence showing that the responses of the neurons are not modulated by context. In particular, in all cases where we did not obtain a significant difference with the ANOVA test, we evaluated whether the responses across the two stories were statistically equivalent or whether the result was inconclusive (i.e., that there was a lack of a significant difference but no evidence of equivalent responses). Both for the responsive and non-responsive neurons and both during encoding and recall, in the vast majority of cases (≥95%; see Table S3), we found that the responses to the different stories were statistically equivalent.

To further quantify the population results, for each experimental session we used the firing of all the recorded neurons (i.e., independent of any responsiveness criterion for selecting neurons) to predict the trial label (i.e., which person and which story was presented in each trial) with a naive Bayesian decoder. Figure 5A shows the average decoding performance during encoding. On the left, the 4 × 4 confusion matrix (two stories for each of the two identities) averaged across all sessions is shown, which gave a mean of 39% hits and is above the chance performance (25%). Note, however, that the decoder could not properly distinguish between the stories within a given identity. To further quantify these results, we ran two independent decoders—the first one decoding only the identity (irrespective of the story) and the second one decoding the story (irrespective of the identity). In line with the results described with the 4 × 4 decoder, the decoding of the identity gave an 84% average performance (across sessions), and the decoding of the story gave a 46% average performance, which is close to chance level (50%) (Figure 5A, center panels). The right panels show the surrogate distributions and the observed performance for each experimental session when decoding identity (top) and story (bottom) separately. All sessions led to a significant decoding performance for identity (permutation tests, $p < 0.01$ in all cases), and none of the sessions gave a significant decoding performance for story. Similar results were obtained during the recall phase (Figure 5B). As before, the 4 × 4 decoding performance averaged across sessions was 40%, mixing the fact that it was possible to decode the identity but not the story presented in each trial. However, when decoding only the stimulus identity, the average performance was 75%, with 9 out of 14 sessions showing a significant performance ($p < 0.01$, and in one case $p = 0.02$), and none of the sessions showed a significant decoding performance for the story (with an average performance of 45%). Finally, Figure 5C depicts the time-resolved decoding performance during the encoding (left) and recall (right) phases. During encoding, we observed that the decoding of stimulus identity increases at about 300 ms after the stimulus onset and peaks at about 500 ms, whereas the decoding of the story remains at chance level, in agreement with the previous results. This was also confirmed with a permutation test that identified time periods in which identity decoding and the difference between identity and story decoding performances were significantly above chance. In no time period was the decoding performance for the story significantly larger than chance. Similarly, during recall, the decoding for identity increases above the chance level at about 2,500 ms before the subject named the relevant stimulus, peaking between 1 s before and after the recall onset, whereas, again, the decoding of the story remained at chance level. In summary, using the activity of all the recorded neurons, the decoding results are similar to the ones obtained with the ANOVA, permutation, and equivalence tests using the responsive (and then the non-responsive) neurons, thus emphasizing that the non-responsive neurons did not contribute information about the story presented either in the encoding or in the recall task.

DISCUSSION

We have implemented a paradigm in which subjects learned and recalled stories mimicking simple episodic memories, to test whether human MTL single-neuron responses are modulated by the context (e.g., two different stories involving the same person) and/or the task performed by the subjects (passively viewing the pictures of the persons/places involved in the stories, encoding and recalling the stories). Patients learned the stories in a few trials, and MTL neurons showed consistent responses after the presentation of the pictures eliciting the neurons' responses (both during VP and encoding), as well as when recalling the specific stories, but in this case, even before they named the particular persons/places triggering the responses of the neurons. The main result of this study is 3-fold.

comparison with the VP phase (the same normalization factor was used for each neuron during encoding and VP). Responses during the recall phase are weaker and smeared in time, but they still show a similar profile for both stories and a clear difference between the responsive and non-responsive identities.

(B) Grand averages of the normalized instantaneous firing rates across all responsive neurons (mean ± sem), showing similar responses for the two stories involving the response-eliciting identity and no responses for the non-responsive identities.

(C) Responses of the same neurons analyzed in (A), during encoding (left) and recall (right) for the two different stories. The panels show a large variability across the population of neurons but, for each neuron, similar responses for the two stories (with significant Pearson correlations).

(D) Responses across tasks for the two stories (joined with a segment) of each of the responsive neurons. The responses were significantly correlated (Pearson) when comparing the VP, encoding, and recall tasks.

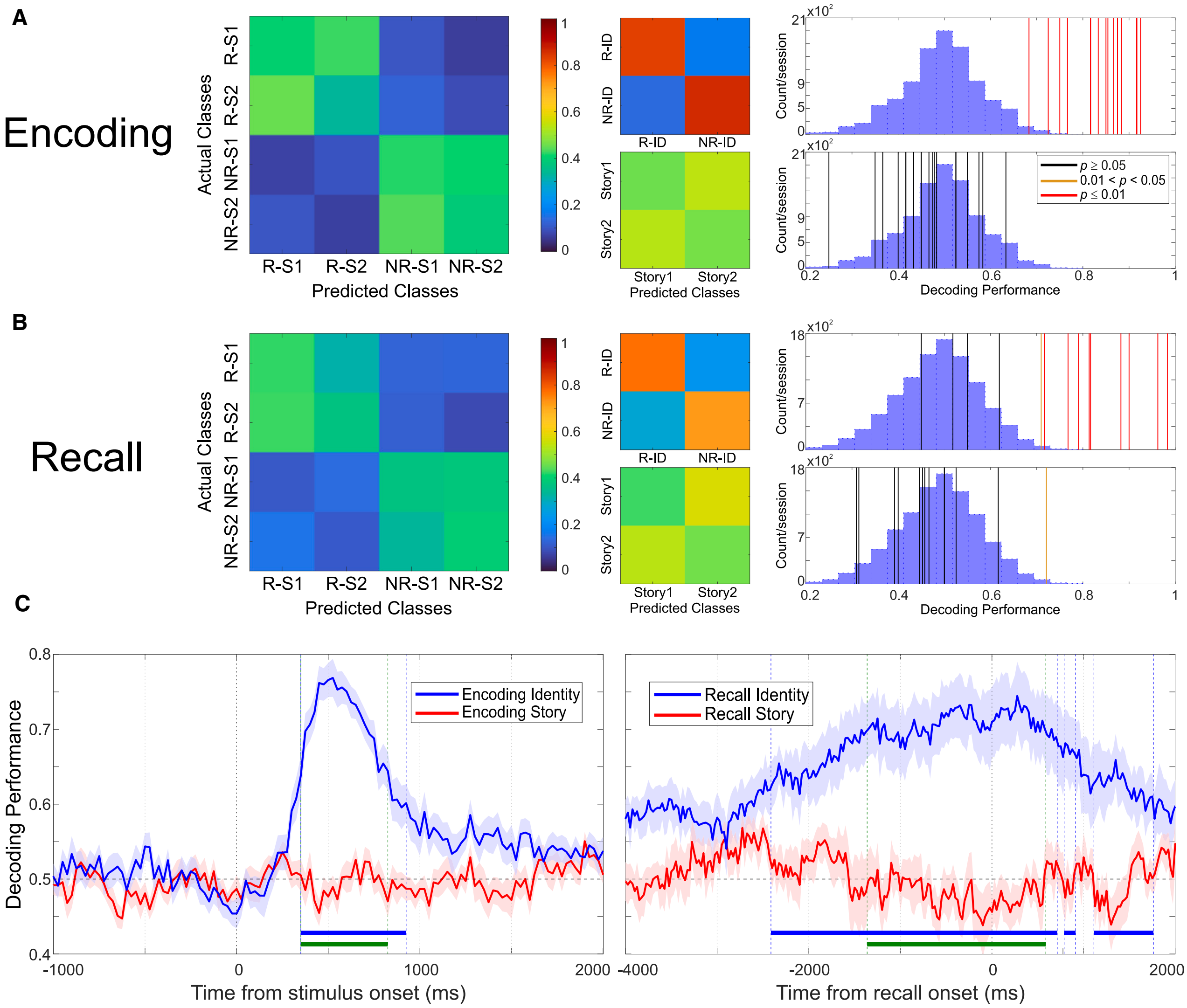

Figure 5. Decoding results from the activity of all recorded neurons

(A) (Left) The average confusion matrix obtained in the encoding phase, when decoding the four classes (left) corresponding to the two stories with the responsive (R-S1 and R-S2) and the two stories with non-responsive stimuli (NR-S1 and NR-S2). The average performance (39%) was greater than chance, but the decoder could not properly distinguish between the stories for each identity. The center panels show the confusion matrices when predicting only the identity irrespective of the story (top), and the story irrespective of the identity (bottom). The decoding of identity was above chance level (84%), whereas the decoding of story was at about chance level (46%). The right panels show the corresponding decoding performances for each individual session for identity (top) and story (bottom).

(B) Same as in (A) but during the recall phase, showing results qualitatively similar to those obtained in the encoding phase.

(C) Time-resolved performance (mean ± SEM) for each decoder (identity/story) during encoding (left) and recall (right). In both cases, there is a clear increase in the decoding of identity, while the one of story remains at chance levels. This was confirmed with a permutation test ($p < 0.001$; i.e., the observed values were always to the right of the 1,000 surrogate distributions), where time periods of significant identity decoding (blue) and difference between identity and story decoding performances (green) were found, but not for story-decoding performance.

First, in the vast majority of cases (97% during encoding and 100% during recall), we found no context modulation of the MTL responses—in other words, a neuron firing to a particular person/place showed strong but not statistically different responses to two different stories involving the same person/place, both during encoding and recall. Second, in most cases, the neurons consistently responded to the same persons/places during the visual perception and memory tasks; 94% of the neurons responded significantly to the same persons/places during passive viewing and memory encoding, and 73% of the neurons responded also during recall. This latter difference can be attributed to the fact that recall involves internally generated activations that may in some cases fail to fully activate the neuronal assembly encoding a particular person/place. Third, MTL neurons did not show conjunctive coding—in other words, besides the lack of context and task modulation mentioned in the previous points, almost none of the initially non-responsive neurons started responding to combinations

of persons/places in specific stories or tasks (Figure S7). Non-responsive neurons did not carry conjunctive information about the context, which we verified with a decoding approach using all the recorded neurons.

As is typically said, "absence of evidence is not evidence of absence." However, the lack of context modulations and conjunctive coding described here cannot be attributed to a specific statistical criterion because consistent results were obtained using ANOVA, permutation tests on the strength and latency of the responses, and a decoding approach considering all the recorded neurons. Furthermore, results were also similar when considering different criteria of statistical significance (Table S2). In addition, using equivalence tests, we found that in most cases the neuronal responses in the different contexts were statistically equivalent (Table S3). Most important, the lack of context and task modulations shown in our data contrasts markedly with the ubiquitous findings of conjunctive coding, as well as context and task modulations in the rodent and monkey hippocampus (see reviews[1–3,8,11]), and with two previous works that reported conjunctive coding with human single-neuron recordings.[34,35] However, in one of these works the activations of the neurons during encoding and recall were compared in the one-trial learning paradigm,[34] and it is unclear how reliable (in time) such coding is and how the corresponding memories are eventually stored in the long term. The other study involved a perceptual discrimination task (not directly varying context),[35] in which differences between the responses to pictures of the same person were reported (depending on whether the picture was a target or a lure image in an oddball-like paradigm), though the responses to the target and lure images were not statistically compared (see Box 1 in Quian Quiroga[36]).

Our results also contrast with the large evidence of context modulations in human hippocampal responses reported in fMRI studies[16–29]—some of them using sequences of item presentations to manipulate context and the related episodic information,[18–20,23,26] similar to our experimental paradigm. However, it should be noted that, in spite of general similarities, our paradigm is different from the ones reported in previous fMRI studies because, for example, remembering a sequence of initially unrelated items, involves different cognitive demands compared to remembering four items linked by a narrative that mimics a simple episodic memory, as in our case, given that the narrative provides a general context or story that facilitates its recall. Given these differences, our results should not be necessarily generalized to previous fMRI works, but considering that with fMRI it is not possible to measure directly the activity of single neurons, it is plausible to speculate that rather than a modulation of the responses at the single-cell level, as found in rodents and monkeys, the context modulation observed at the voxel level with fMRI in humans can be attributed to the coactivation of different assemblies, each of them encoding a particular person or place in a context-independent way; for example, the memories of meeting a person in two different places involve the activation of the same assembly coding for the person together with the assemblies coding for one or the other place. In other words, our results are in line with the large amount of evidence demonstrating a critical role of the hippocampus for context-dependent memories,[1,2,5,8,15,16,29] but we argue that this is implemented differently at the single-cell level in humans, with coactivations of context-independent assemblies, rather than context modulations of the responses of individual neurons.

These results suggest that compared to other species, a different principle underlies the coding of memories in the human MTL, from which it seems plausible to speculate that such unique coding may in turn underlie human cognitive abilities. Besides some specific differences, there are large similarities between the human, monkey, and rodent hippocampi[37,38] and it is in principle surprising that a different principle applies to humans. However, it has been argued that the inputs to the human hippocampus are different due to the use of language, which gives later responses to the meaning of the stimulus (rather than the stimulus itself).[30] In this respect, the presence of context-independent assemblies shown here might be ideal, among others, for metacognition (to think about thoughts irrespective of any particular context or circumstances), as well as for inferences and generalizations, establishing relationships across contexts.

Limitations of the study

The main result presented here contrasts with the vast literature showing context modulation of single-cell responses in monkeys and rodents. It also contrasts with works inferring analogous context modulations from fMRI responses in humans. Our initial goal was not to directly compare with these studies, and further experiments using the same (or analogous) paradigms should be performed to quantitatively compare the degree of context modulations across species and observed with different recording techniques. However, it is remarkable that in our case, using a paradigm emulating simple naturalistic memories with varying contexts, we did not observe context modulations at the single-cell level. Moreover, in our study, we used familiar identities, and future experiments should elucidate whether a similar lack of context modulation is observed for novel unfamiliar ones as well. Furthermore, it could in principle be argued that the lack of context modulation that we found in our study is given by the fact that, during encoding, we used the same pictures of specific identities in both stories. Note, however, that we obtained similar results during recall, where the subjects repeated the stories as they remembered them, without seeing the response-eliciting pictures.

The recording of individual neurons in awake and behaving human subjects allows the extraordinary opportunity to study the neural underpinnings of cognitive and, particularly, memory processes in humans. There are, however, limitations compared to human fMRI studies due to the relatively limited availability of patients and the number and coverage of the recordings, which is exclusively determined by the clinical criteria. There are also limitations compared to animal studies due to clinical and ethical considerations, and particularly due to the fact that in our recordings we cannot identify specific hippocampal subfields. However, the ubiquity of our findings suggests that a lack of context modulation at the single-neuron level might be common in the hippocampus and amygdala, something that should be explored further in future experiments.

RESOURCE AVAILABILITY

Lead contact

Further information and requests for resources should be directed to the lead contact, Rodrigo Quian Quiroga (rqqg1@le.ac.uk).

Materials availability

This study did not generate new unique reagents.

Data and code availability

- Responses observed in the current dataset have been shared (see key resources table).
- All original code has been made publicly available as of the date of publication (see key resources table).
- Any additional information required to reanalyze the data reported in this paper is available from the lead contact upon request.

ACKNOWLEDGMENTS

The authors thank all patients for their participation and the hospital's staff for technical assistance. This work was supported by grants from the Medical Research Council (G1002100), the Biotechnology and Biological Sciences Research Council (BB/T001291/1), the Human Frontiers Science Project, the Royal Society (IEC\R2\170302), and CONICET (PID 53, 2012).

AUTHOR CONTRIBUTIONS

Conceptualization, R.Q.Q., H.G.R., and T.I.P.; data curation, H.G.R., T.I.P., L.G., and R.Q.Q.; formal analysis, H.G.R. and T.I.P.; funding acquisition, R.Q.Q., H.G.R., M.P.R., and S.K.; investigation, H.G.R., T.I.P., F.J.C., L.G., A.N., S.C., F.N., A.V., G.A., and R.Q.Q.; methodology, R.Q.Q., H.G.R., and T.I.P.; resources, R.Q.Q., H.G.R., S.K., and M.P.R.; software, H.G.R., F.J.C., and R.Q.Q.; supervision, R.Q.Q., H.G.R., and S.K.; visualization, H.G.R.; writing – original draft, H.G.R. and R.Q.Q.; writing – review & editing, all authors.

DECLARATION OF INTERESTS

The authors declare no competing interests.

STAR★METHODS

Detailed methods are provided in the online version of this paper and include the following:

- KEY RESOURCES TABLE
- EXPERIMENTAL MODEL AND STUDY PARTICIPANT DETAILS
- METHOD DETAILS
  - Screening and experimental paradigm
- QUANTIFICATION AND STATISTICAL ANALYSIS
  - Spike sorting, responsiveness criteria, and statistical analyses
  - Equivalence test
  - Population analyses and grand average plots
  - Decoding analysis

SUPPLEMENTAL INFORMATION

Supplemental information can be found online at https://doi.org/10.1016/j.celrep.2024.115218.

Received: November 21, 2023
Revised: November 13, 2024
Accepted: December 26, 2024
Published: January 15, 2025

REFERENCES

1. Eichenbaum, H., and Cohen, N.J. (2014). Can we reconcile the declarative memory and spatial navigation views on hippocampal function? Neuron *83*, 764–770.
2. Eichenbaum, H., Dudchenko, P., Wood, E., Shapiro, M., and Tanila, H. (1999). The hippocampus, memory, and place cells: is it spatial memory or a memory space? Neuron *23*, 209–226.
3. Rolls, E.T., and Wirth, S. (2018). Spatial representations in the primate hippocampus, and their functions in memory and navigation. Prog. Neurobiol. *171*, 90–113.
4. Markus, E.J., Qin, Y.L., Leonard, B., Skaggs, W.E., McNaughton, B.L., and Barnes, C.A. (1995). Interactions between location and task affect the spatial and directional firing of hippocampal neurons. J. Neurosci. *15*, 7079–7094.
5. Smith, D.M., and Mizumori, S.J.Y. (2006). Learning-related development of context-specific neuronal responses to places and events: the hippocampal role in context processing. J. Neurosci. *26*, 3154–3163.
6. Wood, E.R., Dudchenko, P.A., and Eichenbaum, H. (1999). The global record of memory in hippocampal neuronal activity. Nature *397*, 613–616.
7. Wood, E.R., Dudchenko, P.A., Robitsek, R.J., and Eichenbaum, H. (2000). Hippocampal neurons encode information about different types of memory episodes occurring in the same location. Neuron *27*, 623–633.
8. Shapiro, M.L., Kennedy, P.J., and Ferbinteanu, J. (2006). Representing episodes in the mammalian brain. Curr. Opin. Neurobiol. *16*, 701–709.
9. Rolls, E.T., Xiang, J., and Franco, L. (2005). Object, space, and object-space representations in the primate hippocampus. J. Neurophysiol. *94*, 833–844.
10. Baraduc, P., Duhamel, J.-R., and Wirth, S. (2019). Schema cells in the macaque hippocampus. Science *363*, 635–639.
11. Moser, E.I., Moser, M.-B., and McNaughton, B.L. (2017). Spatial representation in the hippocampal formation: a history. Nat. Neurosci. *20*, 1448–1464.
12. Frank, L.M., Brown, E.N., and Wilson, M. (2000). Trajectory encoding in the hippocampus and entorhinal cortex. Neuron *27*, 169–178.
13. Pastalkova, E., Itskov, V., Amarasingham, A., and Buzsáki, G. (2008). Internally generated cell assembly sequences in the rat hippocampus. Science *321*, 1322–1327.
14. Squire, L.R., and Zola-Morgan, S. (1991). The medial temporal lobe memory system. Science *253*, 1380–1386.
15. Moscovitch, M., Rosenbaum, R.S., Gilboa, A., Addis, D.R., Westmacott, R., Grady, C., McAndrews, M.P., Levine, B., Black, S., Winocur, G., and Nadel, L. (2005). Functional neuroanatomy of remote episodic, semantic and spatial memory: a unified account based on multiple trace theory. J. Anat. *207*, 35–66.
16. Leal, S.L., and Yassa, M.A. (2018). Integrating new findings and examining clinical applications of pattern separation. Nat. Neurosci. *21*, 163–173.
17. Yassa, M.A., and Stark, C.E.L. (2011). Pattern separation in the hippocampus. Trends Neurosci. *34*, 515–525.
18. Kumaran, D., and Maguire, E.A. (2006). The dynamics of hippocampal activation during encoding of overlapping sequences. Neuron *49*, 617–629.
19. Koolschijn, R.S., Emir, U.E., Pantelides, A.C., Nili, H., Behrens, T.E.J., and Barron, H.C. (2019). The Hippocampus and Neocortical Inhibitory Engrams Protect against Memory Interference. Neuron *101*, 528–541.e6.
20. Hsieh, L.-T., Gruber, M.J., Jenkins, L.J., and Ranganath, C. (2014). Hippocampal activity patterns carry information about objects in temporal context. Neuron *81*, 1165–1178.
21. Milivojevic, B., Varadinov, M., Vicente Grabovetsky, A., Collin, S.H.P., and Doeller, C.F. (2016). Coding of Event Nodes and Narrative Context in the Hippocampus. J. Neurosci. *36*, 12412–12424.
22. Chadwick, M.J., Hassabis, D., and Maguire, E.A. (2011). Decoding overlapping memories in the medial temporal lobes using high-resolution fMRI. Learn. Mem. *18*, 742–746.

23. Berron, D., Schütze, H., Maass, A., Cardenas-Blanco, A., Kuijf, H.J., Kumaran, D., and Düzel, E. (2016). Strong Evidence for Pattern Separation in Human Dentate Gyrus. J. Neurosci. *36*, 7569–7579.

24. Kyle, C.T., Stokes, J.D., Lieberman, J.S., Hassan, A.S., and Ekstrom, A.D. (2015). Successful retrieval of competing spatial environments in humans involves hippocampal pattern separation mechanisms. Elife *4*, e10499.

25. Davachi, L., Mitchell, J.P., and Wagner, A.D. (2003). Multiple routes to memory: distinct medial temporal lobe processes build item and source memories. Proc. Natl. Acad. Sci. USA *100*, 2157–2162.

26. Libby, L.A., Reagh, Z.M., Bouffard, N.R., Ragland, J.D., and Ranganath, C. (2019). The Hippocampus Generalizes across Memories that Share Item and Context Information. J. Cognit. Neurosci. *31*, 24–35.

27. Favila, S.E., Chanales, A.J.H., and Kuhl, B.A. (2016). Experience-dependent hippocampal pattern differentiation prevents interference during subsequent learning. Nat. Commun. *7*, 11066.

28. Ballard, I.C., Wagner, A.D., and McClure, S.M. (2019). Hippocampal pattern separation supports reinforcement learning. Nat. Commun. *10*, 1073.

29. Davachi, L. (2006). Item, context and relational episodic encoding in humans. Curr. Opin. Neurobiol. *16*, 693–700.

30. Quian Quiroga, R. (2019). Plugging in to Human Memory: Advantages, Challenges, and Insights from Human Single-Neuron Recordings. Cell *179*, 1015–1032.

31. Rey, H.G., Ison, M.J., Pedreira, C., Valentin, A., Alarcon, G., Selway, R., Richardson, M.P., and Quian Quiroga, R. (2015). Single-cell recordings in the human medial temporal lobe. J. Anat. *227*, 394–408.

32. Gelbard-Sagiv, H., Mukamel, R., Harel, M., Malach, R., and Fried, I. (2008). Internally Generated Reactivation of Single Neurons in Human Hippocampus During Free Recall. Science *322*, 96–101.

33. Ison, M.J., Quian Quiroga, R., and Fried, I. (2015). Rapid Encoding of New Memories by Individual Neurons in the Human Brain. Neuron *87*, 220–230.

34. Kolibius, L.D., Roux, F., Parish, G., Ter Wal, M., Van Der Plas, M., Chelvarajah, R., Sawlani, V., Rollings, D.T., Lang, J.D., Gollwitzer, S., et al. (2023). Hippocampal neurons code individual episodic memories in humans. Nat. Human Behav. *7*, 1968–1979.

35. Suthana, N.A., Parikshak, N.N., Ekstrom, A.D., Ison, M.J., Knowlton, B.J., Bookheimer, S.Y., and Fried, I. (2015). Specific responses of human hippocampal neurons are associated with better memory. Proc. Natl. Acad. Sci. USA *112*, 10503–10508.

36. Quian Quiroga, R. (2021). How Are Memories Stored in the Human Hippocampus? Trends Cognit. Sci. *25*, 425–426.

37. Tapia-González, S., Insausti, R., and DeFelipe, J. (2020). Differential expression of secretagogin immunostaining in the hippocampal formation and the entorhinal and perirhinal cortices of humans, rats, and mice. J. Comp. Neurol. *528*, 523–541.

38. David, A., and Pierre, L. (2006). In The Hippocampus Book, P. Andersen, R. Morris, D. Amaral, T. Bliss, and J. O'Keefe, eds. (Oxford University Press). https://doi.org/10.1093/acprof:oso/9780195100273.003.0003.

39. Davis, T.S., Caston, R.M., Philip, B., Charlebois, C.M., Anderson, D.N., Weaver, K.E., Smith, E.H., and Rolston, J.D. (2021). LeGUI: A Fast and Accurate Graphical User Interface for Automated Detection and Anatomical Localization of Intracranial Electrodes. Front. Neurosci. *15*, 769872.

40. Chaure, F.J., Rey, H.G., and Quian Quiroga, R. (2018). A novel and fully automatic spike-sorting implementation with variable number of features. J. Neurophysiol. *120*, 1859–1871.

41. Brainard, D.H. (1997). The Psychophysics Toolbox. Spatial Vis. *10*, 433–436.

42. Quiroga, R.Q., Reddy, L., Kreiman, G., Koch, C., and Fried, I. (2005). Invariant visual representation by single neurons in the human brain. Nature *435*, 1102–1107.

43. Viskontas, I.V., Quiroga, R.Q., and Fried, I. (2009). Human medial temporal lobe neurons respond preferentially to personally relevant images. Proc. Natl. Acad. Sci. USA *106*, 21329–21334.

44. Rey, H.G., De Falco, E., Ison, M.J., Valentin, A., Alarcon, G., Selway, R., Richardson, M.P., and Quian Quiroga, R. (2018). Encoding of long-term associations through neural unitization in the human medial temporal lobe. Nat. Commun. *9*, 4372.

45. Holt, G.R., Softky, W.R., Koch, C., and Douglas, R.J. (1996). Comparison of discharge variability in vitro and in vivo in cat visual cortex neurons. J. Neurophysiol. *75*, 1806–1814.

46. Rutishauser, U., Ye, S., Koroma, M., Tudusciuc, O., Ross, I.B., Chung, J.M., and Mamelak, A.N. (2015). Representation of retrieval confidence by single neurons in the human medial temporal lobe. Nat. Neurosci. *18*, 1041–1050.

47. Kamiński, J., Brzezicka, A., Mamelak, A.N., and Rutishauser, U. (2020). Combined Phase-Rate Coding by Persistently Active Neurons as a Mechanism for Maintaining Multiple Items in Working Memory in Humans. Neuron *106*, 256–264.e3.

48. Rey, H.G., Gori, B., Chaure, F.J., Collavini, S., Blenkmann, A.O., Seoane, P., Seoane, E., Kochen, S., and Quian Quiroga, R. (2020). Single Neuron Coding of Identity in the Human Hippocampal Formation. Curr. Biol. *30*, 1152–1159.e3.

49. Quiroga, R.Q., Mukamel, R., Isham, E.A., Malach, R., and Fried, I. (2008). Human single-neuron responses at the threshold of conscious recognition. Proc. Natl. Acad. Sci. USA *105*, 3599–3604.

50. Lakens, D. (2017). Equivalence Tests: A Practical Primer for t Tests, Correlations, and Meta-Analyses. Soc. Psychol. Personal. Sci. *8*, 355–362.

51. Maris, E., and Oostenveld, R. (2007). Nonparametric statistical testing of EEG- and MEG-data. J. Neurosci. Methods *164*, 177–190.

STAR★METHODS

KEY RESOURCES TABLE

REAGENT or RESOURCE	SOURCE	IDENTIFIER
Deposited data		
Spiking activity for the responses in the current dataset	This paper	https://www.mcw.edu/departments/neurosurgery/research/rey-laboratory/software-and-datasets
Software and algorithms		
LeGUI toolbox	Davis et al.[39]	https://github.com/Rolston-Lab/LeGUI
MATLAB	MathWorks	https://www.mathworks.com
Wave_clus	Chaure et al.[40]	https://github.com/csn-le/wave_clus
Psychophysics Toolbox version 3	Brainard[41]	www.psychtoolbox.org/
Custom-built MATLAB code	This paper	https://doi.org/10.5281/zenodo.14260488
Text Aloud v3.0	NextUp Technologies	https://nextup.com/

EXPERIMENTAL MODEL AND STUDY PARTICIPANT DETAILS

We report results from 21 experimental sessions in 9 patients with pharmacologically intractable epilepsy (one left-handed, six females, 18–52 years old). The recordings were performed both at King's College Hospital in London (UK) and "Hospital El Cruce" in Buenos Aires (Argentina), where patients were implanted with chronic depth electrodes for 7–10 days to determine the seizure focus.[30,31] All patients gave their written informed consent to participate in this study, which was approved by King's College Hospital Research Ethics Committee or "Hospital El Cruce" Medical Institutional Review Board. At each trajectory, the surgeon implanted a clinical probe (Behnke-Fried from AdTech) and the associated micro inner bundle that had a total of nine microwires at its end, eight active recording channels and one (low impedance) reference. The electrodes were implanted bilaterally in the hippocampus and amygdala, with each patient receiving between 1 and 4 probes. Electrode locations were based exclusively on clinical criteria and were verified by CT co-registered to preoperative MRI (using the LeGUI toolbox[39]; see electrode localization and examples in Figure S2). The recordings were performed with a Blackrock Microsystems device, and the differential signal obtained from each channel (with respect to the local low impedance reference) was filtered between 0.3 and 7,500 Hz, and sampled at 30,000 Hz. During their stay in the hospital, each subject performed between 1 and 4 sessions of the experimental paradigm described here.

METHOD DETAILS

Screening and experimental paradigm

As in previous works,[31,42] a simple visual task was used to identify stimuli triggering responses in the recorded neurons. A standard laptop running the Psychophysics Toolbox (www.psychtoolbox.org/,[41]) under MATLAB (https://www.mathworks.com) was used for stimulus presentation. Subjects were sitting facing the laptop where a set of over 100 pictures were presented for 500 m, 15 times each, in pseudorandom order and in sequences lasting between 30 and 60 s. In two patients the pictures were displayed for 1,000 m, but results were similar to those showing the pictures for 500 m. The set of pictures used in these 'screening sessions' included items familiar to the patient, such as images of celebrities, landmarks, and the relatives and friends of the patients, as these tend to trigger MTL neuron responses.[43]

The data from the screening sessions was quickly analyzed offline to select two items that elicited responses in different channels (i.e., in different neurons), which were used in the experiment reported here. This way, an item that was considered responsive in one channel was non-responsive, and used as control, for the channel where the other response was found. In cases where only one response was present, we randomly chose a second (non-responsive) item that was used as control. For each of the two selected items, two different stories, reflecting two simple episodic memories in very different contexts, were generated. All four stories had a similar structure, namely, 'when', 'where', 'who' and 'what'. For example (see Figure 1, story 3): "Last year, a strange event took place on Christmas …" (temporal context; when); "I was traveling around and I ended up visiting the Iguazu Waterfalls …" (spatial context; where); "then, out of nowhere, I spotted Jackie Chan …" (a person; who); "after chatting for a while he told me he liked my colorful shirt" (action context; what). Each of the four stories had approximately the same length and involved similar type of stories, but with different contexts (see Figure 1). This way, we had a design with which we could compare the responses of the neurons to the two different identities, and to the two different stories per identity. In 16 out of 21 sessions, the selected items to be studied were persons, and in the remaining 5 cases the selected items were specific places, which was the same for the two associated stories but with different people and identical places, leading to qualitatively similar results (see examples in Figures S3–S6).

The stories were presented as sequences of 4 pictures on the laptop screen (Figure 1). Each picture was presented for 1,000 ms at the center of the screen, and separated between 2.65 and 2.9 s from each other, during which time, the sentence referring to the picture (see example above) was read by a computer synthetized voice (with each sentence across stories having the same duration). For the first four patients, the stories were read by the subjects before the picture presentations, but we then switched to the audio-visual presentation (the sequence of pictures with the audio telling the stories) because it was more comfortable for the patients. Since we did not observe differences in the type of responses, we have pulled together the data of the first four patients with the rest.

On each trial, the four stories were presented in pseudorandom order, with a [5, 5.1] sec blank in between and starting with a fixation cross presented for [0.8, 1] sec. Subjects were asked to remember the stories presented during these 'encoding trials', which were later followed by the 'recall trials', in which the subjects saw the temporal context picture presented for 1 s and had 12 s to verbally recall the associated story. The subjects' verbal recalls were recorded with a microphone and input to an analog auxiliary channel in the Blackrock data acquisition system, synchronized to the neuronal recordings. From the microphone recordings we identified the timestamps when the subjects started to mention the relevant stimuli for that story and identified that as the recall onset. From the 21 experimental sessions, we were only able to process the recall in 14 of them. This is because there were technical issues to record the microphone stream in 5 sessions, and another 2 sessions had to be discarded as the recall performance was too poor (less than 10 correct trials for any given identity). This trial structure (encoding-recall of the 4 stories) was repeated between 10 and 15 times, a number of trials that we have previously shown to be sufficient to evaluate whether the neuronal responses in two conditions are statistically the same or different.[44] A recall trial was considered correct if the subject was able to retrieve the story (place, person, and action) upon seeing the cue associated with a given temporal context used at the beginning of each story. Subjects managed to correctly learn the different stories after a few trials as measured by the recall performance (percentage of correctly recalled stories; Figure 1B).

In addition, to confirm that the neurons were still responsive to the stimuli chosen in the previous screening sessions, we ran a simple visual presentation task (VP) before and after the experimental paradigm (10–15 trials each), using the same structure of the screening sessions but with the 14 stimuli used during the experimental paradigm (see Figure 1). Since VP responses were similar before and after the experimental paradigm, we pulled them together for further analyses.

QUANTIFICATION AND STATISTICAL ANALYSIS

Spike sorting, responsiveness criteria, and statistical analyses

The collected data were processed offline, and the high-frequency activity (between 300 and 3,000 Hz) was extracted to identify the spikes of the recorded neurons. Spike detection and sorting was done with Wave_clus.[40] For each isolated unit, we computed different metrics to evaluate the sorting quality (Figure S1). Specifically, we computed the percentage of inter spike intervals (ISI) that were smaller than 3ms, the firing rate (across the whole recording session), and the following metrics:

$$SNR_{peak} = \frac{|\overline{\mathbf{W}}(peak)|}{\sigma_{\mathbf{W}}(1)}$$

the peak signal to noise ratio, defined as the ratio of the absolute value of the mean waveform at the peak timepoint against the standard deviation of the waveforms at the first sample epoched;

$$SNR_{whole} = \sqrt{\frac{1}{N}\sum_{i=1}^{N}\left(\frac{\overline{\mathbf{W}}(i)}{\sigma_{\mathbf{W}}(i)}\right)^2}$$

the SNR across the whole waveform, defined as the root-mean-square of the vector generated by the pointwise ratio between the mean waveform and the standard deviation waveform (with $N = 64$ being the number of points on each waveform);

$$CV2 = \frac{1}{M-1}\sum_{i=1}^{M-1}\frac{|\mathrm{ISI}(i+1) - \mathrm{ISI}(i)|}{\frac{\mathrm{ISI}(i+1)+\mathrm{ISI}(i)}{2}}$$

the modified coefficient of variation,[45] where M is the number of spikes detected on a given unit.

We also quantified the distribution of units isolated per wire. Overall, the quality is comparable to other studies,[46,47] with very few violations of the refractory period or small SNR values. As in these previous studies, we excluded from the dataset all the isolated units that had a firing rate smaller than 0.1 Hz.

For the encoding trials, we looked for a response latency onset for the selected stimuli in each of the 4 different stories, as we have done in previous works.[44,48] The latency was defined as the upwards crossing of the instantaneous firing rate over a threshold for at least 90 m, with short periods of less than 20 m going below threshold being disregarded. The instantaneous firing rate was calculated by convolving the spike train with a Gaussian kernel with σ = 10 m (truncated at 1% amplitude). The threshold was set to the mean plus 4 standard deviations, computed across all stimuli between 900 m and 100 m before stimulus onset (with a minimum at 5 Hz, for neurons with low baseline firing). Then, we calculated the number of spikes in a 500 m

window from the latency onset, and when it was not possible to define a latency onset with the criterion mentioned above, we used a fixed window between 200 m and 700 m from stimulus onset. We defined the strength of the response as the median of the spike count across trials. Given the design of our experiment, analyzing responses to two identities in two contexts each, we considered a neuron to be responsive during the encoding phase if a two-way ANOVA of the spike count, with factors identity and context, led to $p < 0.01$, and the response eliciting stimuli had a well-defined latency onset for at least one story. From a total of 318 recorded channels in the hippocampus (194) and amygdala (124), we were able to isolate 737 units across all experimental sessions (see Table S1 for a split across regions for each session). Applying the response criterion to these units led to a set of 33 being responsive for identity during the encoding phase (14 from amygdala and 19 from hippocampus). Given that we were interested in any possible modulation of the neuronal responses by the different contexts/stories, we considered the modulation by story to be significant if either the factor context or the interaction of the ANOVA was significant. For the responses in the VP task, we implemented a similar criterion, but in this case using a one-way ANOVA with factor identity. We did not find different results for amygdala and hippocampus, so we presented the results pooled across these regions (see examples in Figures S3–S6).

As in previous works,[49] neurons were classified into single- or multi-units based on: (i) the spike shape and the variance of the cluster; (ii) the SNR_{peak} (namely, the ratio between the peak value of the waveform and the standard deviation, typically larger than about 5); (iii) the ISI distribution of each cluster; and (iv) the presence of a refractory period for the single units, i.e., about <1% spikes with an ISI smaller than 3 m. This way, from a total of 737 units, 384 were classified as single units and from the 33 units that showed a significant response during the encoding, 30 were classified as single units. In Figure S1B we recomputed the quality metrics for each subpopulation of multi and single units, showing improved metrics in the SU population.

For the recall task, given that it has been shown that the neural responses during recall start before naming the identity eliciting the neurons' responses and have longer durations and larger jitter than during picture viewing,[32,33] we counted spikes in a window [-1500,500] ms from recall onset. To avoid false positives, and since we could not define a proper latency response onset as during the encoding phase, we required the median firing rate in the response window to be larger than the mean plus s.d. during the baseline period (before the context cue), with a minimum of 3 Hz, for at least one of the stories. Then, as in the encoding phase, we applied a two-way ANOVA on the spike count (factors identity and context) and defined significance with $p < 0.01$. As we were only able to evaluate the neural activity during recall for 14 out of the 21 sessions, in this case we isolated 554 neurons (174 from amygdala and 380 from hippocampus), from 238 recorded channels (71 from amygdala and 167 from hippocampus). Out of the 33 units being responsive for identity during the encoding phase, 22 were analyzed during the recall phase (6 from amygdala and 16 from hippocampus).

To further validate the results, for all the recorded neurons we performed a permutation test of the pairwise comparison of the strength of spike response for each identity across stories.[44,48] Specifically, we compared their absolute difference in strength of response to a distribution of 1000 surrogate values, created by randomly permuting the trial labels for the responses to the two stories. Similarly, we also compared latencies during the encoding phase (in this case only for the responsive neurons, since we could not properly define a latency for the non-responsive ones). For this, we calculated the absolute difference in latency of response for each response pair and, as above, for each test the p-value was obtained by comparing the observed statistic (for strength or latency) against 1000 surrogate values.

In all cases, similar results were obtained when using slightly different statistical criteria (see Table S2), thus showing that results were not due to the choice of a specific criterion for comparing responses.

Equivalence test

Since in most cases our results show a lack of statistical differences, we complemented the analysis with an equivalence test developed in the field of pharmacokinetics (e.g., to test if a new cheaper drug performs as well as an existing one).[50] In particular, we used a two-one-sided tests (TOST) approach,[50] where two composite null hypothesis are tested on specified equivalence bounds: $H0_1 \Delta \le \Delta_L$ and $H0_2 \Delta \ge \Delta_U$. When both hypotheses are rejected, it can be argued that the effect falls within the bounds and the differences are equivalent. Specifically, the TOST approach uses the Welch's t tests[50]:

$$t_L = \frac{\mu_1 - \mu_2 - \Delta_L}{\sqrt{\frac{\sigma_1^2}{n_1} + \frac{\sigma_2^2}{n_2}}} \quad \text{and} \quad t_U = \frac{\mu_1 - \mu_2 - \Delta_U}{\sqrt{\frac{\sigma_1^2}{n_1} + \frac{\sigma_2^2}{n_2}}},$$

with μ_i, σ_i and n_i denoting the mean, standard deviation and sample size for each dataset (i.e., the number of trials for each story). The bounds Δ_i were defined in a standardized way based on Cohen's d, $\Delta_i = d_i \times \sigma$, where σ is the pooled standard deviation:

$$\sigma = \sqrt{\frac{(n_1 - 1)\sigma_1^2 + (n_2 - 1)\sigma_2^2}{n_1 + n_2 - 2}}.$$

As previously suggested,[50] we defined symmetric bounds $-d_L = d_U = d_0$ (with a given Type I (α) and II (β) errors; see Table S3 for the different sets of values used) following the normal approximation of the power of equivalence tests, leading to:

$$d_0 = \sqrt{\frac{2\left(z_\alpha + z_{\beta/2}\right)^2}{\min(n_1, n_2)}},$$

where z_p is the inverse of the standard normal cumulative distribution function evaluated at the probability value p.

Population analyses and grand average plots

In the population analyses in Figures 4A and 4B, for each response the instantaneous firing rate was normalized by subtracting the mean baseline activity and dividing by the peak firing rate of the responsive stimuli during encoding (applied to encoding and VP) or recall. This way, the instantaneous firing rate ranged between 0 and 1. In Figures 4C and 4D, we computed the activity for each neuron by subtracting the average firing rate of the responsive and non-responsive stimuli in the corresponding time window (based on the results from Figure 4B, encoding/VP from 100 to 800 m, recall from −2500 to 750 m).

Decoding analysis

For the decoding analysis (Figure 5), for each trial we computed average firing rates across the same time windows as for the ANOVA tests (for VP and encoding, a 500 m window from the latency onset, or from 200 m after stimulus onset when it was not possible to define a latency; for recall, [-1500,500] ms from recall onset). For each session, we input the activity of all the recorded neurons (range = [10,70], mean = 35, std = 20) to a Naive Bayesian decoder with leave-one-out cross-validation, in order to predict which of the four stories (or just the identity, or the story across identities) was presented (or recalled) each time. We evaluated the significance of the decoding performance with 10,000 surrogates, by shuffling trial labels. We also performed a time-resolved decoding analysis, where we used a sliding window (25 m resolution) with a 250 m width for encoding, and a 1000 m width for recall. To evaluate significance in this case we used a permutation test.[51] Specifically, we first computed sign tests at each time bin to assess the difference of performance against chance for identity and story encoding/recall, respectively. A cluster of time bins was defined when the tests were significant ($p < 0.01$) for at least 100ms. We did similarly to evaluate differences between identity and story decoding performance with a paired sign test. Then, we computed the sum of the sign statistic within each cluster, and compared that to the surrogate distribution of the sum of 1000 permutations (shuffling the story labels used on each experimental session). If the observed value was larger than all the surrogates, we could conclude that the p for the test was smaller than 0.001.

Cell Reports, Volume *44*

Supplemental information

Lack of context modulation in human single neuron responses in the medial temporal lobe

Hernan G. Rey, Theofanis I. Panagiotaropoulos, Lorenzo Gutierrez, Fernando J. Chaure, Alejandro Nasimbera, Santiago Cordisco, Fabian Nishida, Antonio Valentin, Gonzalo Alarcon, Mark P. Richardson, Silvia Kochen, and Rodrigo Quian Quiroga

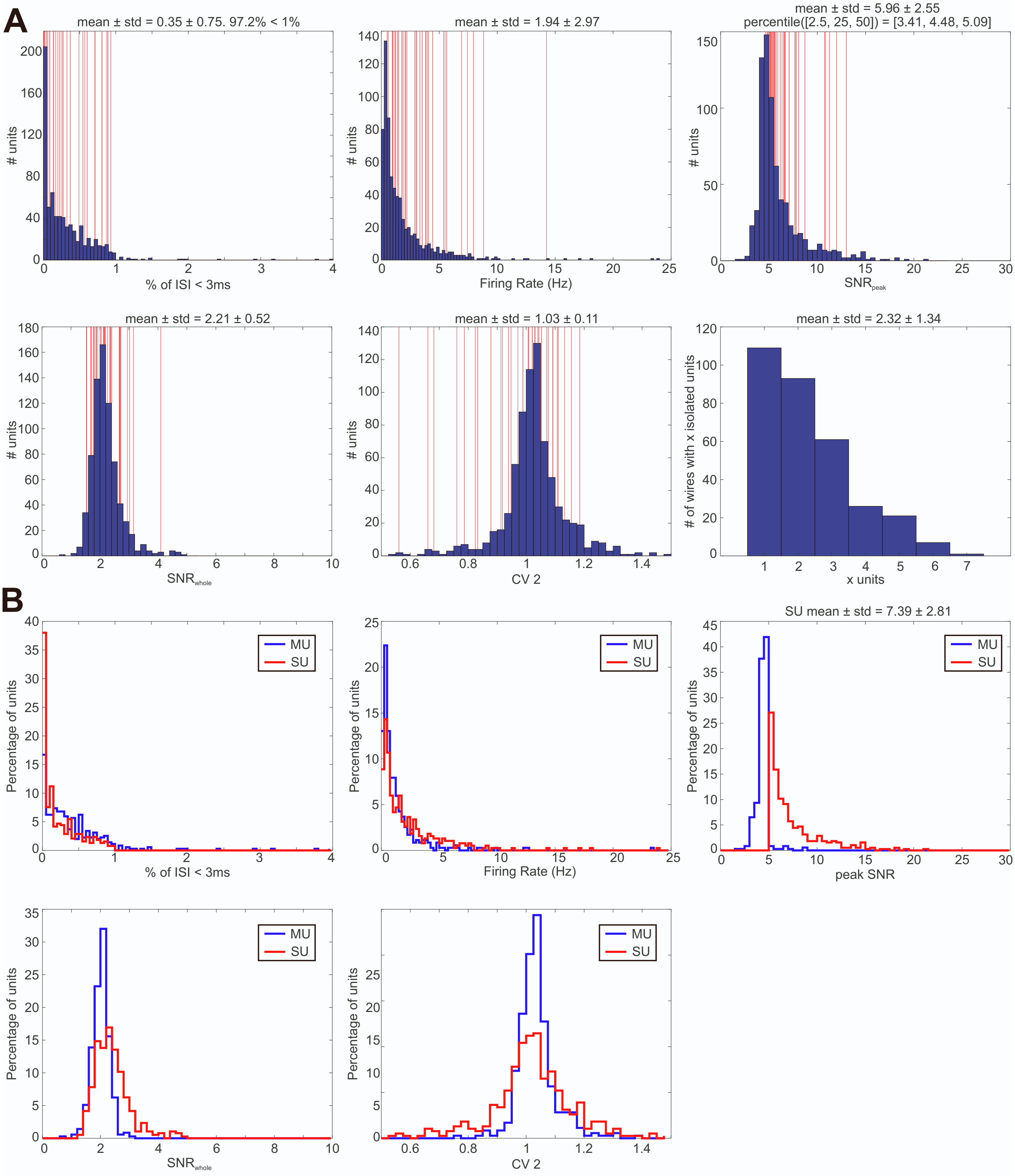

Figure S1. Spike sorting quality metrics. (A) For all the units isolated in the dataset we computed the percentage of ISI that were less than 3ms, the firing rate (over the whole recording session), the SNR_{peak} (using the peak amplitude of the mean waveform), the SNR_{whole} (considering the SNR across the whole waveform), the modified coefficient of variation, and the distribution of isolated units per wire (see Methods for all the specific definitions of the metrics). Vertical lines denote the individual values for the 33 units that showed significant identity responses during the encoding phase of the paradigm. (B), Same as before but the distributions were calculated separately for single (SU) and multi (MU) unit populations.

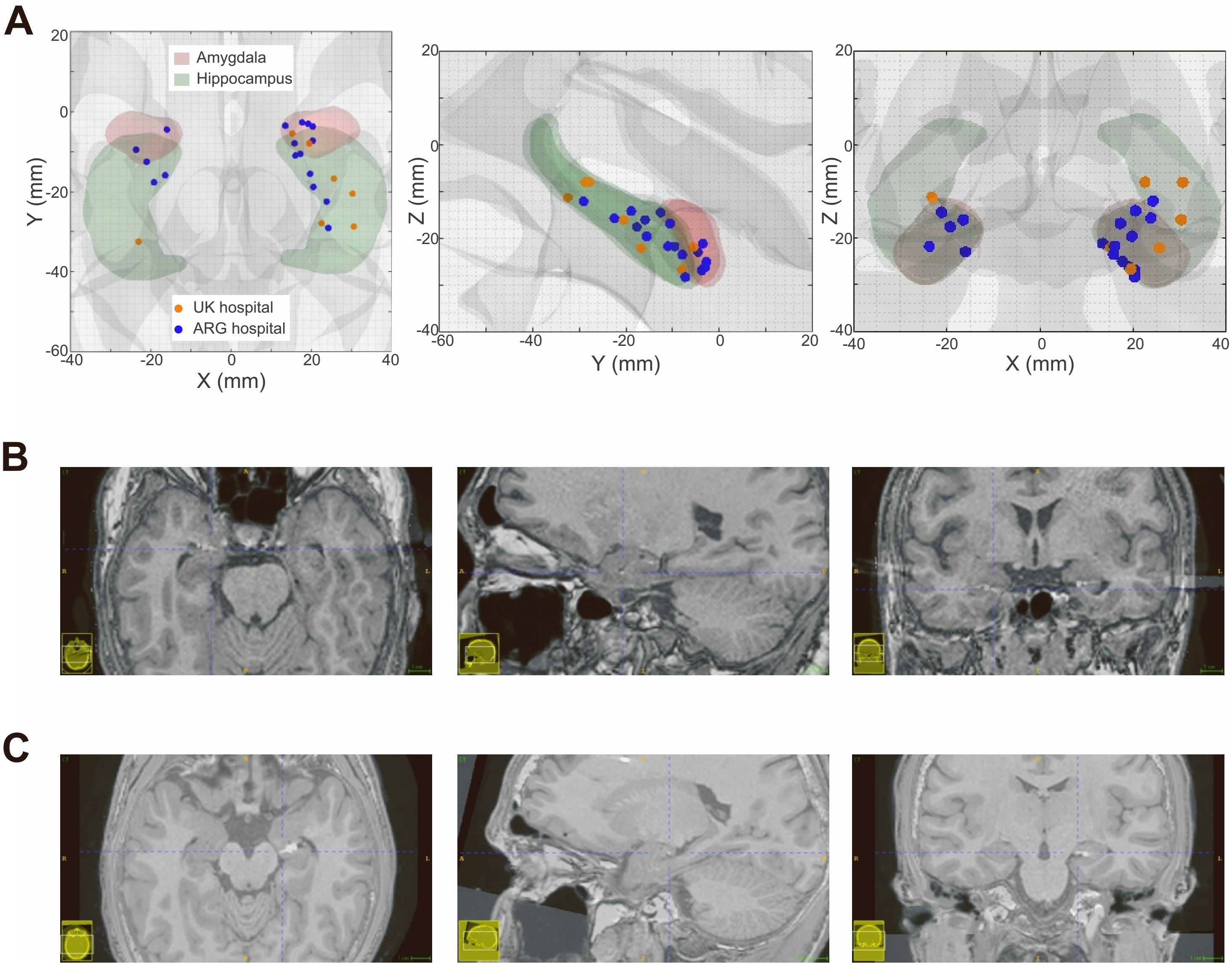

Figure S2. Electrode implantation. (A), microelectrode localization for all the probes included in the current dataset (color coded according to the hospitals where they were implanted). Hippocampus and amygdala are shown in green and red, with 15 and 9 probes reaching the respective structures. From left to right, each panel shows axial, sagittal, and coronal views. (B), CT-MRI coregistration showing an example of a probe reaching the right amygdala. Note the artefact in the CT generated by the microwire bundle. (C), Same as (B), but for a probe reaching the left hippocampus.

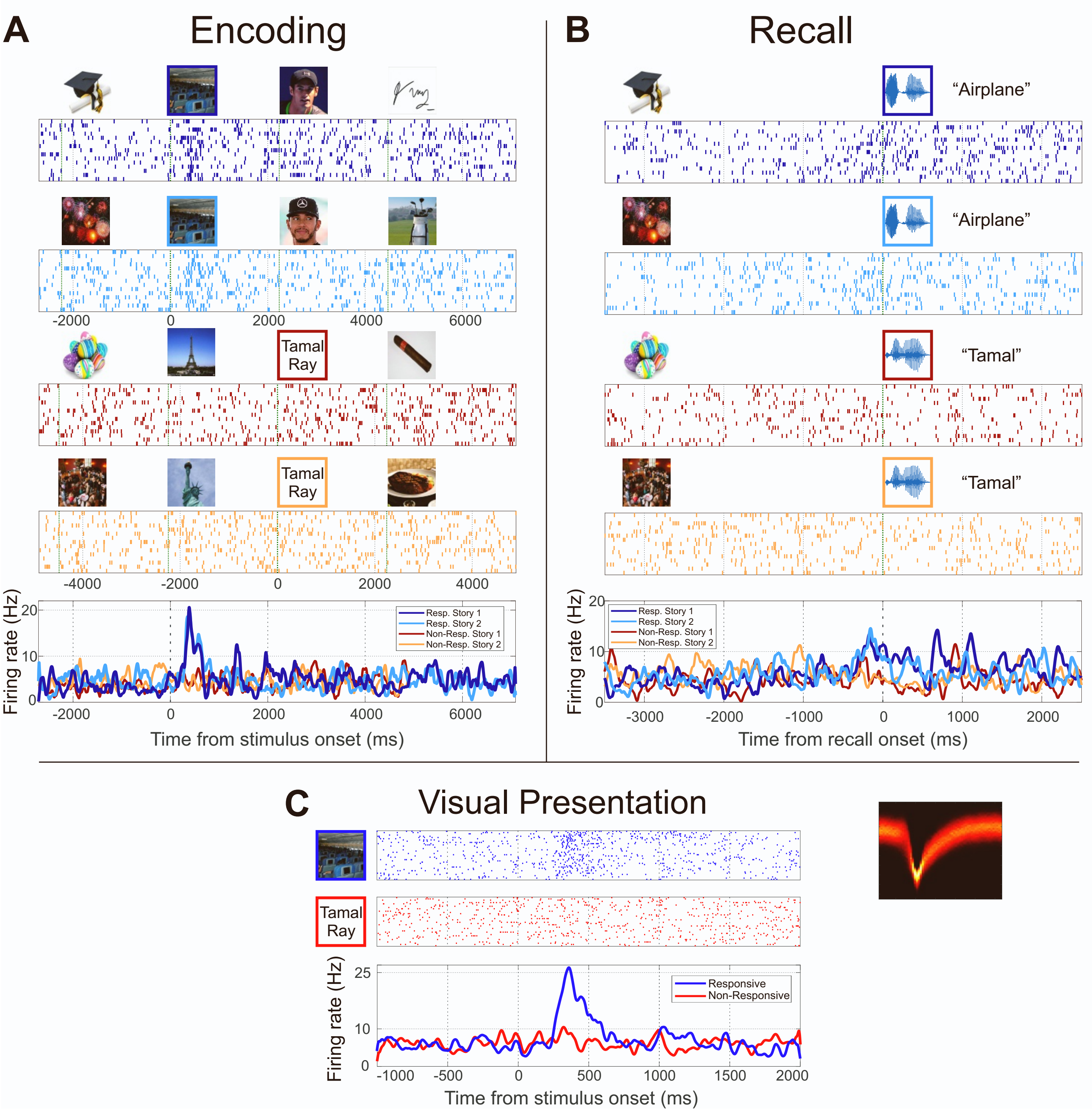

Figure S3. Exemplary neuron responding to a place. Hippocampal neuron responsive to a picture of an *airplane* cabin, but not to *Tamal* (a contestant in the TV show *British Bake Off*), during encoding (A), recall (B), and visual presentation (VP) (C). A density plot of the waveforms associated with the putative single neuron is also shown. The identity effect in the ANOVA test was significant during encoding ($p \sim 10^{-8}$, total df = 59), recall ($p \sim 10^{-5}$, total df = 58), and VP ($p \sim 10^{-5}$, total df = 59). There was no significant effect for the story both during encoding (p = 0.45) and recall (p = 0.33). Copyright notes: Picture of Andy Murray was cropped from "2015 Australian Open - Andy Murray 6" by Brendan Dennis, licensed under CC BY-SA 4.0. Picture of Lewis Hamilton was cropped from "Lewis Hamilton 2016 Malaysia 2" by Morio, licensed under CC BY-SA 4.0.

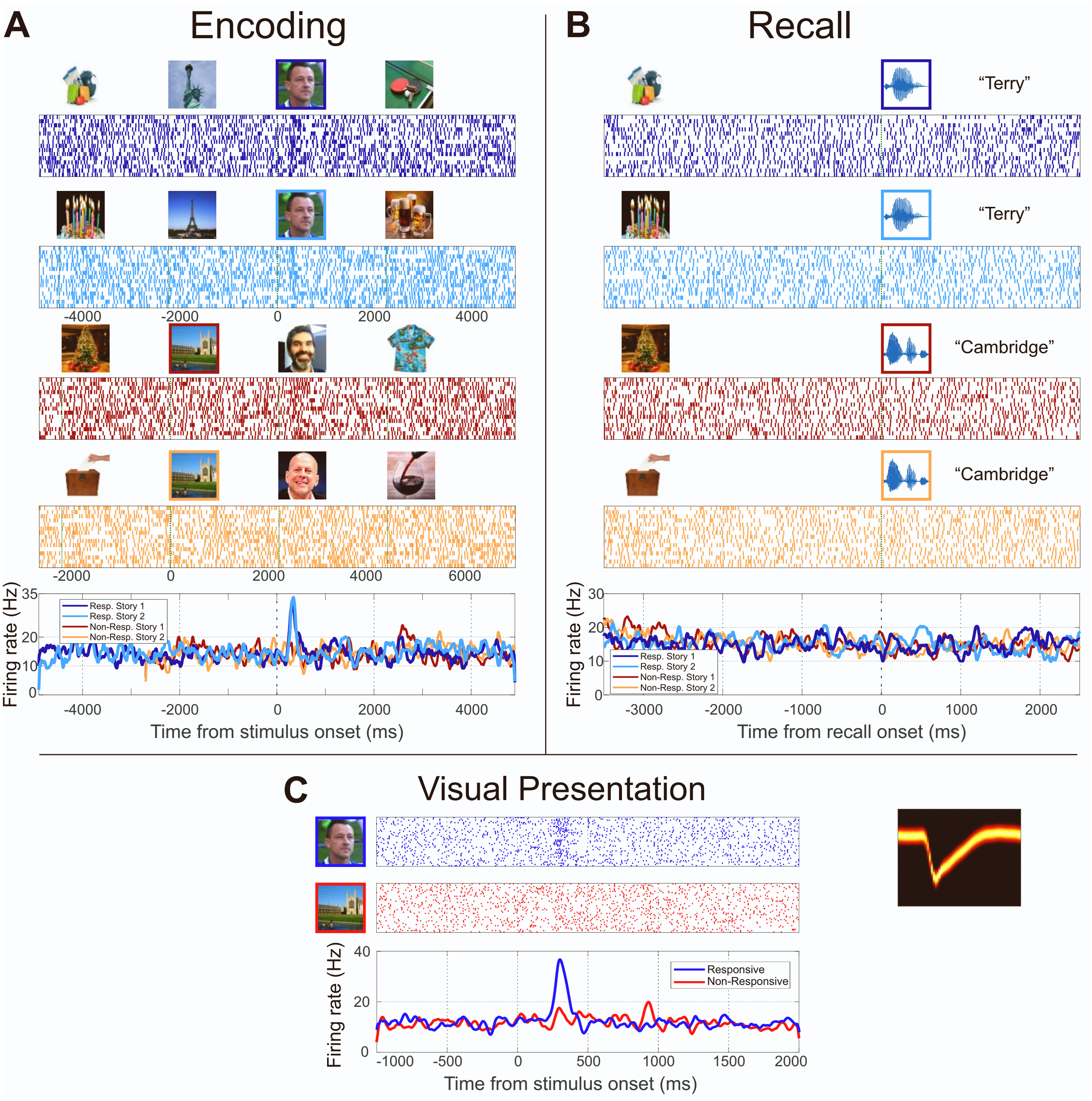

Figure S4. **Exemplary neuron that responded during encoding and VP, but not during recall**. Hippocampal neuron that responded to *John Terry* (former captain of Chelsea FC), but not to the *King's College Chapel at the University of Cambridge*, during encoding ($p \sim 10^{-6}$, total df = 59) (A) and VP ($p \sim 10^{-3}$, total df = 79) (C), but not during recall (p = 0.23, total df = 59) (B). There was no significant effect for the story both during encoding (p = 0.1) and recall (p = 0.18). A density plot of the waveforms associated with the putative single neuron is also shown. Copyright notes: Picture of John Terry was cropped from "Chelsea 5 Sunderland 1 (34821048206)" by @cfcunofficial, licensed under CC BY-SA 2.0. Picture of Bruce Willis was cropped from "Bruce Willis (4840565824)" by Gage Skidmore, licensed under CC BY-SA 2.0. Picture of Dr Antonio Valentin (co-author of the paper) is a self-portrait.

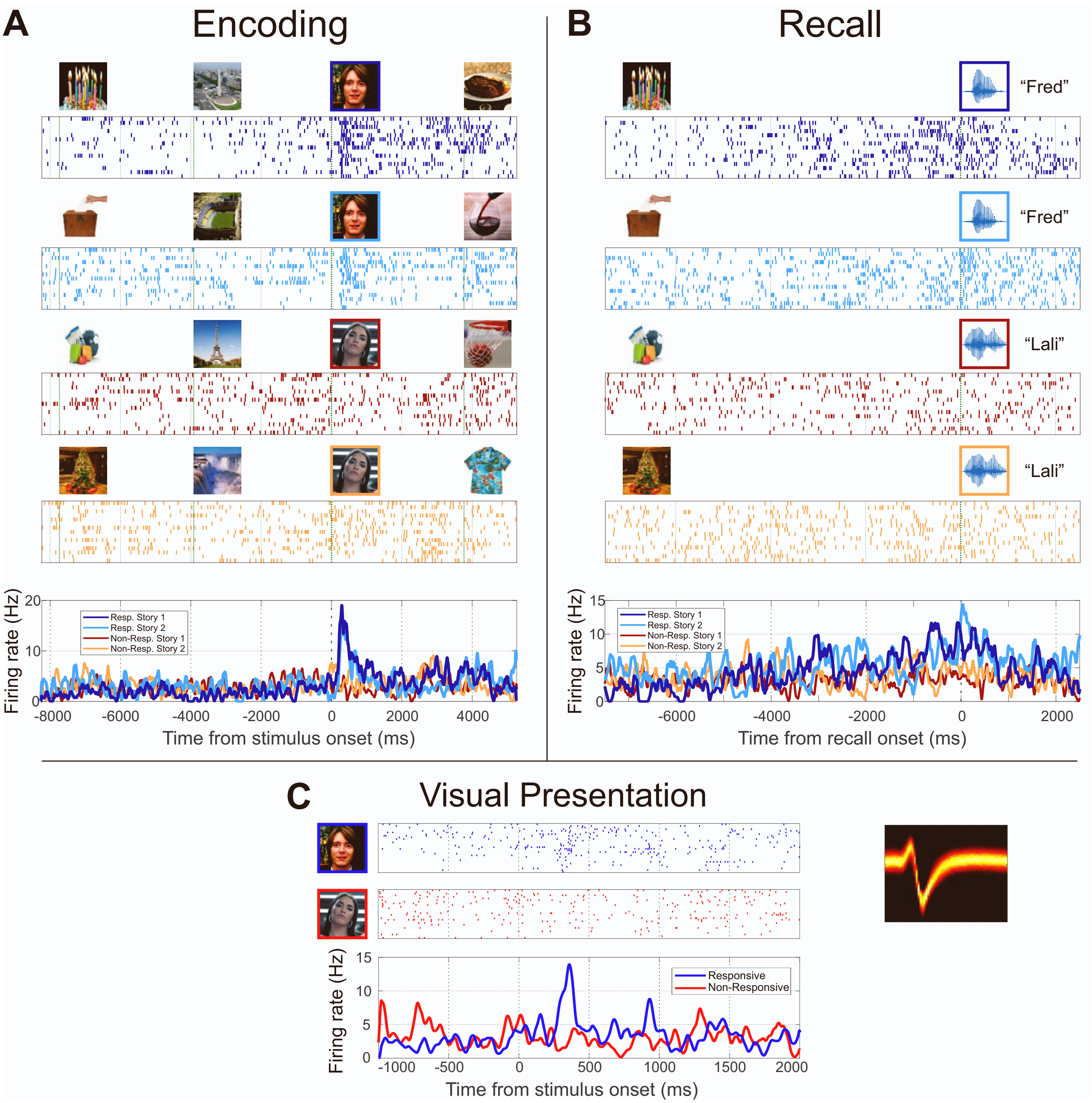

Figure S5. **Exemplary neuron that responded during encoding, VP and recall**. Hippocampal neuron with a response to *Fred Weasley* (a character from the movie *Harry Potter*), but not to *Lali Esposito* (a singer from Argentina), during encoding ($p \sim 10^{-10}$, total df = 59) (A), recall ($p \sim 10^{-8}$, total df = 58) (B), and VP ($p \sim 10^{-3}$, total df = 59) (C). There was no significant effect for the story both during encoding ($p = 0.07$) and recall ($p = 0.014$). A density plot of the waveforms associated with the putative single neuron is also shown.

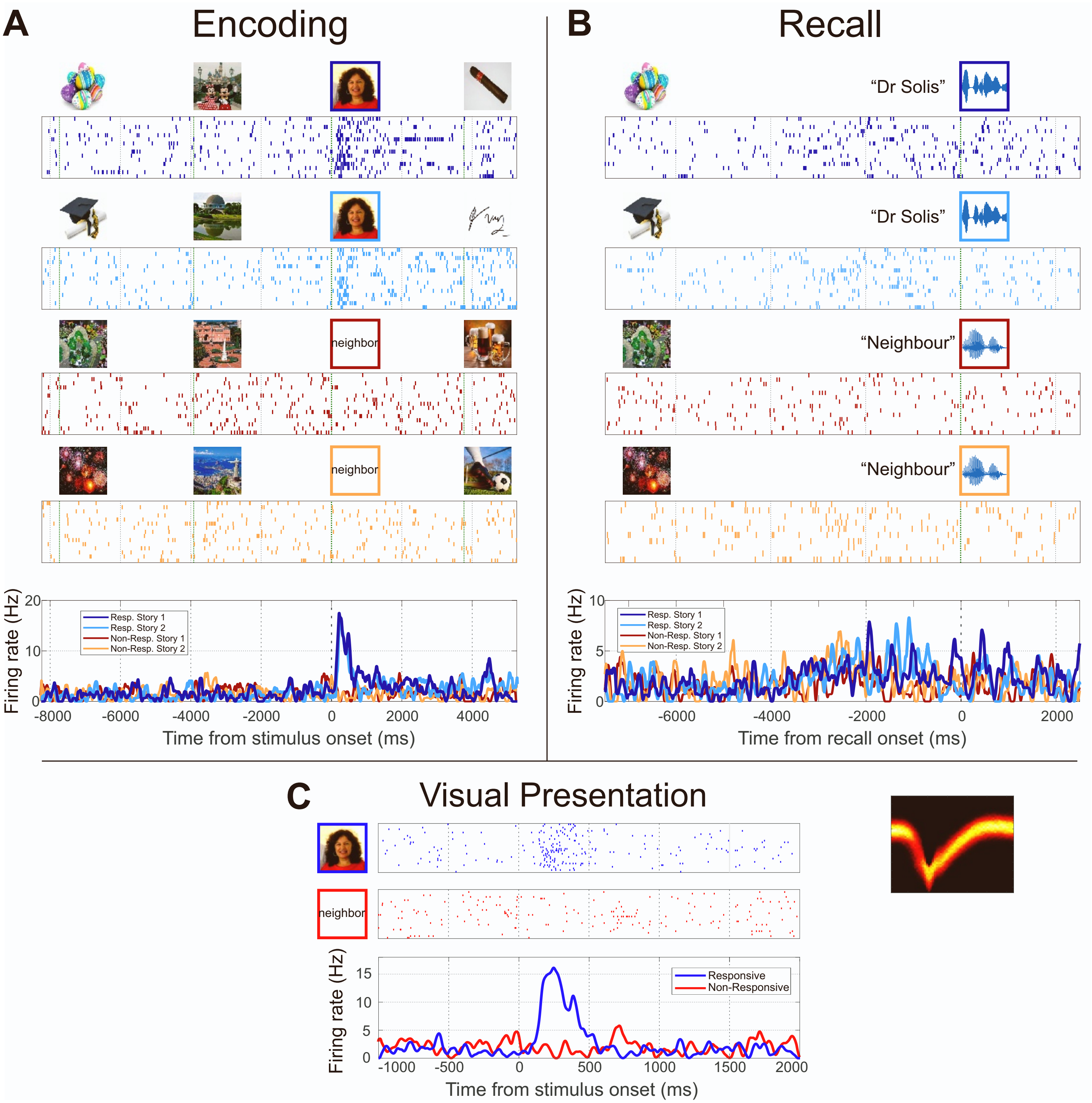

Figure S6.Exemplary neuron from the amygdala that responded during encoding, VP and recall. The neuron shows a response to *Dr Patricia Solis* (a neuropsychologists that was part of the clinical team treating the patient during their hospital stay), but not to the *picture of the patient's neighbor*, during encoding (p ~10^{-12}, total df = 59) (A), recall (p ~10^{-3}, total df = 53) (B), and VP (p ~10^{-10}, total df = 59) (C). There was no significant effect for the story both during encoding ($p = 0.14$) and recall ($p = 0.57$). A density plot of the waveforms associated with the putative single neuron is also shown. Picture of Dr Patricia Solis (who was part of the clinical team) is a self-portrait.

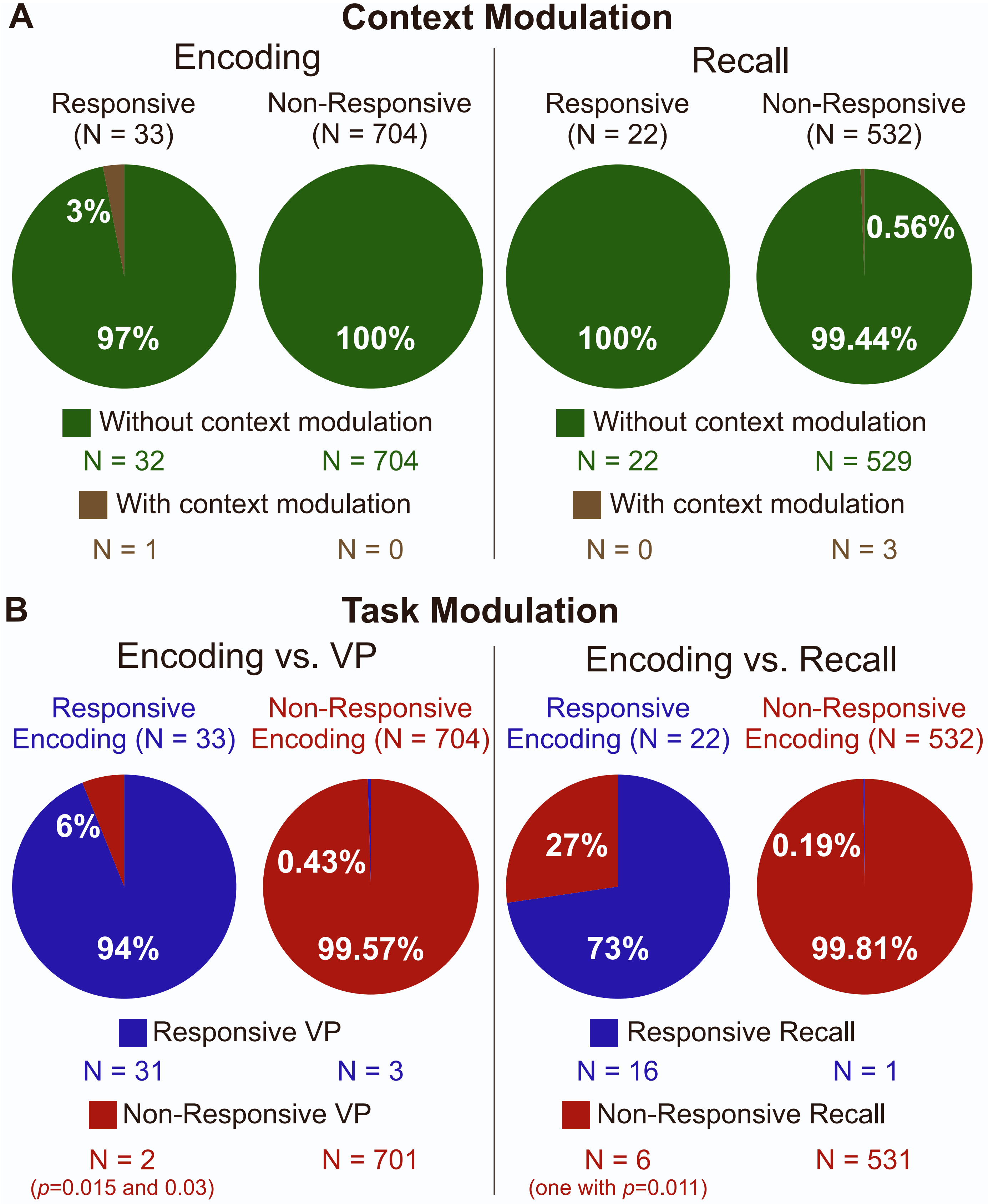

Figure S7. Number of neurons showing context and task modulations based on the ANOVA results. (A), Responsive (left) and non-responsive neurons (right) showing context modulation during encoding and recall. (B), Neurons compared across different tasks. Most of the neurons that responded significantly to a given identity during encoding, also responded to the same identity during the visual presentation task (with two neurons being close to the significance threshold). A large percentage of those neurons also responded during recall (with the activity being driven by an internal process without external stimulation). The vast majority of neurons not eliciting responses during encoding were also not responsive during VP and recall.

Session number	1	2	3	4	5	6	7	8	9	10	11	12	13	14	15	16	17	18	19	20	21	Total
# neurons in Hippocampus	32	42	36	38	14	25	33	10	-	56	37	-	-	-	-	11	10	11	28	27	21	431
# neurons in Amygdala	20	19	13	9	5	-	37	5	17	11	26	23	25	18	18	-	-	-	23	19	18	306
# resp. during encoding in Hippocampus	1	1	2	2	2	1	2	2	0	1	1	0	0	0	0	1	1	2	0	0	0	19
# resp. during encoding in Amygdala	1	0	1	0	0	0	0	0	2	0	0	1	1	1	2	0	0	0	3	1	1	14

Patient id	1	2	3	4	5	6	7	8	9	mean ± std
# resp. per session	2	1.5	2	2	1	1	1.25	1.33	1.66	1.53 ± 0.41

Table S1. Number of units recorded on each area (amygdala and hippocampus) per experimental session. We also show the distribution across experimental sessions of all the 33 neurons eliciting a response during the encoding phase. Sessions 1-4 correspond to patient 1; sessions 5-6 correspond to patient 2 (both of them from the UK); sessions 7-8 correspond to patient 3; sessions 9, 10, and 11 correspond to patient 4, 5 and 6, respectively; sessions 12-15 correspond to patient 7; sessions 16-18 correspond to patient 8; sessions 19-21 correspond to patient 9. To better visualize the contribution of each patient to the dataset, we quantified the average number of observed responsive neurons per session.

	$p < 0.01$	$p < 0.05$
ANOVA test with responsive neurons during encoding (N = 33)	1 [a]	2
ANOVA test with non-responsive neurons during encoding (N = 704)	0	3
ANOVA test with responsive neurons during recall (N = 22)	0	3 [b]
ANOVA test with non-responsive neurons during recall (N = 532)	3	5
Surrogate test on strength with responsive neurons during encoding (N = 33)	0	1
Surrogate test on latency with responsive neurons during encoding (N = 33)	0	0
Surrogate test on strength with non-responsive neurons during encoding (N = 2 x 704 = 1,408)	2	6
Surrogate test on strength with responsive neurons during recall (N = 22)	0	0
Surrogate test on strength with non-responsive neurons during recall (N = 2 x 532 = 1,064)	2	14
Decoding during encoding sessions (N = 21)	0	0
Decoding during recall sessions (N = 14)	0	1

Table S2. Number of cases showing significant effects for context/story using different tests and significant levels. The surrogate tests on the non-responsive set of neurons was applied to each identity separately, leading to a number of cases that was twice the number of neurons. For decoding results, each case was an experimental session. [a] This neuron is shown in **Fig. 2C**. [b] One of these neurons is shown in **Figure S5**.

	$\alpha = \beta = 0.01$	$\alpha = \beta = 0.05$
Statistically equivalent tests on responsive neurons during encoding	32 out of 32 (100%)	31 out of 31 (100%)
Statistically equivalent tests on non-responsive neurons during encoding	1382 out of 1408 (98%)	1333 out of 1402 (95%)
Statistically equivalent tests on responsive neurons during recall	22 out of 22 (100%)	18 out of 19 (95%)
Statistically equivalent tests on non-responsive neurons during recall	1033 out of 1058 (98%)	1003 out of 1054 (95%)

Table S3. Number of cases showing statistically equivalent differences in strength for the different pairs of stories from a given identity. For each pair of stories from a given identity in a neuron that led to non-significant results using the ANOVA test, we evaluated if their difference in strength was statistically equivalent using the TOST approach (see Methods). The test outcome was “statistically equivalent” when the composite hypotheses were rejected at level α.
